# Early corticosteroids are associated with lower mortality in critically ill patients with COVID-19: a cohort study

**DOI:** 10.1186/s13054-020-03422-3

**Published:** 2021-01-04

**Authors:** Pablo Monedero, Alfredo Gea, Pedro Castro, Angel M. Candela-Toha, María L. Hernández-Sanz, Egoitz Arruti, Jesús Villar, Carlos Ferrando, the COVID-19 Spanish ICU Network, Pablo Monedero, Alfredo Gea, Pedro Castro, Angel M. Candela-Toha, Marina Vendrell, Gerard Sánchez-Etayo, Amalia Alcón, Isabel Belda, Mercé Agustí, Albert Carramiñana, Isabel Gracia, Miriam Panzeri, Irene León, Jaume Balust, Ricard Navarro, María José Arguís, María José Carretero, Cristina Ibáñez, Juan Perdomo, Antonio López, Manuel López-Baamonde, Tomás Cuñat, Marta Ubré, Antonio Ojeda, Andrea Calvo, Eva Rivas, Paola Hurtado, Roger Pujol, Nuria Martín, Javier Tercero, Pepe Sanahuja, Marta Magaldi, Miquel Coca, Elena del Rio, Julia Martínez-Ocon, Paula Masgoret, Monserrat Tio, Angel Caballero, Raquel Risco, Raquel Bergé, Lidia Gómez, Nicolás de Riva, Ana Ruiz, Beatriz, Tena, Sebastián Jaramillo, José María Balibrea, Francisco Borja de Lacy, Ana Otero, Ainitze Ibarzabal, Raquel Bravo, Anna Carreras, Daniel Martín-Barreda, Alfonso Jesús Alias, Mariano Balaguer, Jorge Aliaga, Alex Almuedo, Joan Ramón Alonso, Rut Andrea, Gerard Sergi Angelès, Marilyn Arias, Fátima Aziz, Joan Ramon Badía, Enric Barbeta, Toni Torres, Guillem Batiste, Pau Benet, Xavi Borrat, María Borrell, Ernest Bragulat, Inmaculada Carmona, Manuel Castellà, Pedro Castro, Joan Ceravalls, Oscar Comino, Claudia Cucciniello, Clàudia De Deray, Oriol De Diego, Paula De la Matta, Marta Farrero, Javier Fernández, Sara Fernández, Anna Fernández, Miquel Ferrer, Ana Fervienza, María Tallo Forga, Daniel Forné, Clàudia Galán, Andrea Gómez, Eduard Guasch, María Hernández-Tejero, Adriana Jacas, Beltrán Jiménez, Pere Leyes, Teresa López, José Antonio Martínez, Graciela Martínez-Pallí, Jordi Mercadal, Guido Muñoz, José Muñoz, Ricard Navarro, Josep María Nicolás, José Tomás Ortiz, Anna Peiró, Manuel Pérez, Esteban Poch, Margarida Pujol, Eduard Quintana, Bartomeu Ramis, Enric Reverter, Irene Rovira, Pablo Ruiz, Elena Sandoval, Stefan Schneider, Oriol Sibila, Carla Solé, Alex Soriano, Dolors Soy, M. Suárez, Adrián Téllez, Néstor David Toapanta, Antoni Torres, Xavier Urra, César Aldecoa, Alicia Bordell, Silvia Martín, Judith Andrés, Alberto Martínez Ruiz, Gonzalo Tamayo Medel, Iñaki Bilbao Villasante, Fernando Iturri Clavero, Covadonga Peralta Álvarez, Julia T. Herrera Díez, Andrea García Trancho, Iñaki Sainz Mandiola, Carmen Ruano Suarez, Angela Ruiz Bocos, Eneritz Urrutia Izagirre, Pablo Ortiz de Urbina Fernández, Naiara Apodaka López, Leire Prieto Molano, Eunate Ganuza Martínez, Iratxe Vallinas Hidalgo, Karmele de Orte Sancho, Celia González Paniagua, Gemma Ortiz Labrador, Mireia Pérez Larrañaga, Marta López Miguelez, Estíbaliz Bárcena Andrés, Erik Urutxurtu Laureano, Maria Jesús Maroño Boedo, Blanca Escontrela Rodríguez, Aitziber Ereñozaga Camiruaga, Deiene Lasuen Aguirre, Ainhoa Zabal Maeztu, Ane Guereca Gala, Iker Castelo Korro, Andrés Álvarez Campo, Alejandro Carcelen Viana, Alejandro Alberdi Enríquez, Xabier Ormazábal Rementeria, Alberto Sánchez Campos, Rosa Gutiérrez Rico, Pablo Barbier Damborenea, Marta Guerenabarrena Momeñe, Borja Cuesta Ruiz, Alejandro López Rico, Ana Rojo Polo, Covadonga García Grijelmo, Mikel Celorrio Reta, Eneko Martín Arroyo, Leire Artaza Aparicio, Iñaki Ituarte Aspiazu, Ane Igeregi Basabe, Itxaso Merino Julian, Isabel Diaz Rico, Maria Paz Martínez, Ramón Adalia Bartolomé, Luigi Zattera, Irina Adalid Hernandez, Leire Larrañaga Altuna, Aina Serrallonga Castells, Adriana Vílchez Garcia, María Núñez, Lorena Román, Isabel Ramos Delgado, Adela Benítez-Cano Martínez, Mireia Chanzá Albert, Juan Carlos Álvarez García, Luis Aguilera Cuchillo, Sandra Beltrán de Heredia, Jesús Carazo Cordobés, Carlos Alberto García Bernedo, Fernando Escolano Villén, Francisco Javier Redondo Calvo, Rubén Villazala González, Victor Baladron González, Patricia Faba, Omar Montenegro, Natalia Bejarano Ramírez, Sergio Marcos Contreras, Alejandro Garcia Rodríguez, Saleta Rey Vázquez, Cristina Garcia Pérez, Eva Higuera Miguelez, Irene Pérez Blanco, David García Rivera, Ane Martín de la Fuente, Marta Pardo, Vanessa Rodriguez, Unai Bengoetxea, Fernando Ramasco, Sheila Olga Santidrián Bernal, Alvar Santa Cruz Hernando, Antonio Planas Roca, Carlos Figueroa Yusta, Esther García Villabona, Carmen Vallejo Lantero, Eva Patiño Rodriguez, Alvaro Esquivel Toledo, David Arribas Méndez, Mar Orts Rodriguez, Rosa Méndez Hernández, Jesús Nieves Alonso, Inés Imaz Artazcoz, Sonia Expósito Carazo, Carlos Román Guerrero, Elena Rojo Rodríguez, Ricardo Moreno González, Julia Hernando Santos, Jara Torrente Pérez, Esperanza Mata Mena, Manuel José Muñoz Martínez, Enrique Alday Muñoz, Patricia Martin Serrano, Laura Cotter Muñoz, Amadea Mjertan, Diego Gutierrez Martínez, Carmen Rodríguez García, Olaya Alonso Viejo, Juan Alvarez Pereira, Ana Carmona Bonet, Diana Parrado López, Eva de Dios Tomas, Rafael Martín Celemin, María Luisa Meilan Paz, Luis Quecedo Gutiérrez, Noemí Diaz Velasco, Gabriel Martin Hernández, Francisco Garcia del Corral, Gloria Hernandez Arias, David Rodriguez Cuesta, Ana Gómez Rice, Encarna Mateos Sevillano, Natalia Olmos Molpeceres, Beatriz Domínguez, Ana Vázquez Lima, Ángel Candela, Ismael AAcevedo Bambaren, Maria Isabel Albala Blanco, Paloma Alonso Montoiro, Fernando Álvarez Utrera, Juan Avellanosa Esteruelas, Amal Azzam López, Alberto José Balvis Balvis, Tommaso Bardi, María Beltrán Martín, Jacobo Benatar Haserfaty, Alberto Berruezo Camacho, Laura Betolaza Weimer, María del Mar Carbonell Soto, Cristina Carrasco Seral, Cristina Cerro Zaballos, Elizabeth Claros Llamas, Pilar Coleta Orduna, Ingrid P. Cortes Forero, Pascual Agustín Crespo Aliseda, María Angélica de Pablo Pajares, Yolanda Díez Remesal, Trinidad Dorado Díaz, Noemí Echevarría Blasco, María Elena Elías Martín, Javier Felices Triviño, Natalia Fernández López, Cristina Fernández Martín, Natalia Ferreiro Pozuelo, Luis Gajate Martín, Clara Gallego Santos, Diego Gil Mayo, María Gómez Rojo, Claudia González Cibrián, Elena Herrera López, Borja Hinojal Olmedillo, Berta Iglesias Gallego, Sassan Khonsari, María Nuria Mane Ruiz, María Manzanero Arroyo, Ana María Mariscal Ortega, Sara Martín Burcio, María del Carmen Martín González, Ascensión Martín Grande, Jose Juan Martín López, Cecilia Martín Rabes, Marcos Martínez Borja, Nilda Martínez Castro, Adolfo Martínez Pérez, Snejana Matcan, Cristina Medrano Viñas, Lisset Miguel Herrera, Adrián Mira Betancur, María Montiel Carbajo, Javier Moya Moradas, Lorena Muñoz Pérez, Mónica Nuñez Murias, Eva Ordiales González, Óscar Ordoñez Recio, Miguel Ángel Palomero Rodriguez, Diego Parise Roux, Lucia Pereira Torres, David Pestaña Lagunas, Juana María Pinto Corraliza, Marian Prieto Rodrigo, Inmaculada Rodriguez Diaz-Regaño, David Rodriguez Esteban, Víctor Rojas Pernia, Álvaro Ruigómez Saiz, Bárbara Saavedra Villarino, Noemí Samaranch Palero, Gloria Santos Pérez, Jaume Serna Pérez, Ana Belén Serrano Romero, Jesús Tercero López, Carlos Tiscar García, Marta de la Torre Concostrina, Eva María Ureta Mesa, Eva Velasco Olarte, Judith Villahoz Martínez, Raúl Villalaba Palacios, Gema Villanueva García, Cristina Vogel de Medeiros, Soraya Gholamian Ovejero, Marta Vicente Orgaz, Patricia Lloreda Herradon, Cristina Crespo Gómez, Tatiana Sarmiento-Trujillo, Noemí García Medina, María Martínez García, Carles Espinós Ramírez, Nabil Mouhaffel Rivero, Jose Antonio Bernia Gil, Sonsoles Martín, María Victoria Moral, Josefina Galán, Pilar Paniagua, Sergio Pérez, Albert Bainac, Ana Arias, Elsa Ramil, Jorge Escudero, Pablo Monedero, Carmen Cara, Andrea Lara, Elena Mendez Martínez, Jorge Mendoza, Íñigo Rubio Baines, Carmen Sala Trull, Pablo Montero López, Alfredo Gea, Alejandro Montero, Rocío Armero Ibañez, Juan Vicente Llau Pitarch, Fernando Rauer Alcóver, Cristina Álvarez Herreros, Cyntia Sánchez Martín, Lucía López Ocáriz Olmos, Marta Navas Moruno, Fernando García Montoto, M. F. Mirón Rodriguez, Laura Fuentes Coco, Cristina Hernández Gamito, Antonio Barba Orejudo, Luis Gerardo Smith Vielma, Yasmina González Marín, Francisco de Borja Amador Penco, Marta Donoso Domínguez, Silvia Esquivel Ramírez, José Antonio Carbonell, Berta Monleón López, Sara Martínez-Castro, Gerardo Aguilar, María Gestal, Pablo Casas, Angel Outeiro Rosato, Andrea Naveiro Pan, María Alonso Portela, Adrián García Romar, Eva Mosquera Rodríguez, Diego Ruanova Seijo, Pablo Rama Maceiras, Francisco Castro-Ceoane, Esther Moreno López, Sergio Gil, Julia Guillén Antón, Patricia García-Consuegra Tirado, Aurora Callau Calvo, Laura Forés Lisbona, María Carbonell Romero, Belén Albericio Gil, Laura Pradal Jarne, María Soria Lozano, Diego Loscos López, Andrea Patiño Abarca, Javier Pérez-Asenjo, Ángel Díez-Domínguez, Ion Zubizarreta, Jon Ramos, Iosu Fernández, Emilio Maseda, Alejandro Suárez de la Rica, Javier Veganzones, Itziar Insausti, Javier Sagra, Sofía Díaz Carrasco, Ana Montero Feijoo, Julio Yagüe, Ignacio Garutti, Javier Hortal, Patricia Piñeiro, Matilde Piñeiro, Matilde Zaballos, Jamil cedeño, Pablo García-Olivares, Alberto Garriido, Jose Eugenioi Guerrero, Eva Bassas Parga, Carmen Deiros Garcia, Elisenda Pujol Rosa, Ana Tejedor Navarro, Roser Font Gabernet, Maria José Bernat, Meritxell Serra Valls, Cristina Cobaleda Garcia-Bernalt, Jesus Fernanz Anton, Adriana Aponte Sierra, Lucia Gil Gomez, Olaia Guenaga Vaqueiro, Susana Hernandez Marin, Laura Pardo Pinzon, Sira Garcia Aranda, Carlos Briones Orejuela, Edgar Cortes Sánchez, Alejandro Romero Fernández, Esther Fernández SanJosé, Patricia Iglesias Garsabal, Guillermo Isidro Lopez, Ana Vicol, Sara Espejo Malagon, María Sanabra Loewe, Laura Grau Torradeflo, Lourdes Blanco Alcaide, Gloria Buenaventura Sanclemente, Pere Serra Pujol, Gustavo Cuadros Mendoza, Miroslawa Konarska, Fedra Bachs Almenara, Agnieszka Golska, Aleix Carmona Blesa, Arantxa Mas Serra, Javier Ripolles Melchor, Ana Nieto Moreno, Káteri Chao Novo, Sandra Gadín López, Elena Nieto Moreno, Bérénice Gutiérrez Tonal, Elena Lucena de Pablo, Barbara Algar Yañez, Beatriz Vázquez Rivero, Beatriz Nozal Mateo, Marina de Retes, Norma Aracil Escoda, Cristina Gallardo Mayo, Rosa Sanz González, Alicia Ruiz Escobar, Maria Laura Pelegrina López, Marina Valenzuela Peña, David Stolle Dueñas, Ane Abad Motos, Alfredo Abad-Gurumeta, Ana Tirado Errazquin, Elena Sáez Ruiz, Nerea Gómez Pérez, Francisco de Borja Bau González, Cesar Morcillo Serra, Jessica Souto Higueras, Rosario Vicente, Raquel Ferrandis, Silvia Polo Martín, Azucena Pajares Moncho, Ignacio Moreno Puigdollers, Juan Pérez Artacho Cortés, Ana Moret Calvo, Ana Pi Peña, María Catalán Fernández, Marina Varela, Pilar Díaz Parada, Raquel Rey Carlín, Sarra Barreiro Aragunde, María Isabel Forés Chiva, A. Javier Agulló, Antonio Pérez Ferrer, María Galiana, Antoni Margarit, Válerie Mourre del Rio, Eva Heras Muxella, Anna Vidal

**Affiliations:** 1 Department of Anaesthesiology and Intensive Care, Clínica Universidad de Navarra, Pio XII, 36, 31008 Pamplona, Spain; 2 Department of Preventive Medicine and Public Health, Medical School, University of Navarra, Pamplona, Spain; 3 Medical Intensive Care Unit, Hospital Clínic, Institut D’investigació August Pi i Sunyer (IDIBAPS), University of Barcelona, Barcelona, Spain; 4 Department of Anesthesiology and Critical Care, Hospital del Ramón y Cajal, Madrid, Spain; 5 Department of Anesthesiology and Critical Care, Hospital de Cruces, Barakaldo, Vizcaya Spain; 6 Ubikare Technology, Vizcaya, Spain; 7 CIBER de Enfermedades Respiratorias, Instituto de Salud Carlos III, Madrid, Spain; 8 Li Ka Shing Knowledge Institute, St Michael’s Hospital, Toronto, ON Canada; 9 Multidisciplinary Organ Dysfunction Evaluation Research Network, Research Unit, Hospital Universitario Dr. Negrín, Las Palmas de Gran Canaria, Spain; 10 Department of Anesthesiology and Critical Care, Hospital Clínic, Institut D’investigació August Pi i Sunyer, Barcelona, Spain

**Keywords:** COVID-19, Intensive Care Unit, Corticosteroids, Critically ill patient, Cohort study, Outcomes, Ventilator-free days, Mortality

## Abstract

**Background:**

Critically ill patients with coronavirus disease 19 (COVID-19) have a high fatality rate likely due to a dysregulated immune response. Corticosteroids could attenuate this inappropriate response, although there are still some concerns regarding its use, timing, and dose.

**Methods:**

This is a nationwide, prospective, multicenter, observational, cohort study in critically ill adult patients with COVID-19 admitted into Intensive Care Units (ICU) in Spain from 12th March to 29th June 2020. Using a multivariable Cox model with inverse probability weighting, we compared relevant outcomes between patients treated with early corticosteroids (before or within the first 48 h of ICU admission) with those who did not receive early corticosteroids (delayed group) or any corticosteroids at all (never group). Primary endpoint was ICU mortality. Secondary endpoints included 7-day mortality, ventilator-free days, and complications.

**Results:**

A total of 691 patients out of 882 (78.3%) received corticosteroid during their hospital stay. Patients treated with early-corticosteroids (n = 485) had lower ICU mortality (30.3% vs. never 36.6% and delayed 44.2%) and lower 7-day mortality (7.2% vs. never 15.2%) compared to non-early treated patients. They also had higher number of ventilator-free days, less length of ICU stay, and less secondary infections than delayed treated patients. There were no differences in medical complications between groups. Of note, early use of moderate-to-high doses was associated with better outcomes than low dose regimens.

**Conclusion:**

Early use of corticosteroids in critically ill patients with COVID-19 is associated with lower mortality than no or delayed use, and fewer complications than delayed use.

## Background

Coronavirus disease 2019 (COVID-19), caused by the severe acute respiratory syndrome coronavirus 2 (SARS-CoV-2), was first recognized in Wuhan, China, in December 2019 [1]. The antiviral immune response is crucial to eliminate the invading virus. However, an inappropriate response may cause a systemic hyperinflammatory state, producing complications such as acute respiratory distress syndrome (ARDS) and multisystem organ failure [2]. Early treatment of this hyperinflammation may be important for reducing mortality in COVID-19 patients.

Corticosteroids are used to treat several hyperinflammatory syndromes [3]. Early after the outbreak, the World Health Organization (WHO) recommended against the routine use of systemic corticosteroids for treating COVID-19 patients, due to their known side effects and a potential slowing of viral clearance [1]. The RECOVERY trial [4] and six smaller randomized clinical trials have shown improved outcomes in severe COVID-19 patients treated with corticosteroids [4–6]. Consequently, the US National Institutes of Health (NIH) recommends the use of dexamethasone to treat COVID-19 patients requiring supplemental oxygen [7], and the WHO stated a strong recommendation for systemic corticosteroid therapy in patients with severe and critical COVID-19, and a conditional recommendation not to use corticosteroid therapy in patients with non-severe COVID-19 [8]. However, the RECOVERY trial has methodological flaws such as the absence of stratification and incomplete information on multiple factors associated with mortality, which may have caused an imbalance between the control and the corticosteroids treated groups [9]. The WHO meta-analysis [5] also has limitations, with 3 incomplete trials stopped prematurely, and excessive weight of the RECOVERY trial, precluding definitive conclusions [9].

Spain is one of the European countries most affected by the COVID-19 pandemic with a broad experience in the use of corticosteroids in Intensive Care Units (ICU) for patients with ARDS [10]. In a large COVID-19 registry of patients admitted into a network of ICUs, we examined whether early use of corticosteroids decreases all-cause mortality and improves clinically relevant outcomes.

## Methods

### Study design

This is a multicentre, observational study with retrospective analysis of prospectively collected data in consecutive critically ill COVID-19 patients admitted from 12th March to 29th June 2020 into a network of ICUs in 36 hospitals from Spain and Andorra. The study was approved by a referral Ethics Committee (Ethics Committee of Euskadi, Spain) and by all participating hospitals.

### Data source and study population

Following a standardized protocol, site investigators collected data from electronic medical records. We recorded pre-admission and daily data from ICU admission to ICU discharge. Before data analysis, two independent investigators and a statistician screened the database for errors against standardized ranges and contacted site investigators with queries.

All consecutive COVID-19 patients admitted to participating ICUs were considered for study entry if they had: age ≥ 18 years and confirmed SARS-CoV-2 infection from a respiratory tract sample using RT-PCR assay. Exclusion criteria were non-confirmed SARS-CoV-2 infection, patients with no data at baseline, patients with do-not-resuscitate orders, and patients who did not meet the outcomes of death or ICU discharge by 29th June 2020.

### Variables, exposures, and endpoints

We recorded data on demographics and comorbidities according to established definitions (See Additional file 1), laboratory findings, vital signs, severity scores at ICU admission, supportive therapies, and relevant outcomes reported by 29th June 2020. We collected pre-ICU-admission and full data set on the first day of ICU (baseline), and the “worst” values during ICU stay (maximum or minimum, depending on the parameter).

For this study, we established a post hoc cut-off at 48 h after ICU admission, based on the comparison of survivors vs. non-survivors, and because it was a reasonable time for a clinician to decide whether therapy with corticosteroids should be initiated based on the initial ICU evolution and the laboratory results (inflammation and organ failure). So, we classified patients in three groups: (i) patients receiving corticosteroids within the first 48 h (early group); (ii) those receiving corticosteroids after 48 h of ICU admission (delayed group); and (iii) those who never received corticosteroids (never group). The union of delayed and never groups is analysed as non-early group. Other exploratory exposures included the administration of corticosteroids at any time during hospital stay (ever-treated group = early plus delayed use) and no administration at all (never-treated group). We also examined patients receiving low dose of corticosteroids (defined as methylprednisolone < 1 mg/kg/d or dexamethasone < 0.12 mg/kg/d or prednisone < 0.5 mg/kg/d) or receiving moderate-to-high doses (any dose higher than low dose).

The primary endpoint was ICU mortality. We excluded from the analysis patients who died or were discharged within the first 48 h. Secondary endpoints were medical and infectious complications, ventilator-free days, ICU length of stay (LOS), and 7-day mortality.

### Statistical analysis

We aimed to enrol as many patients as possible, with no pre-defined sample size.

We report the values of variables as percentages, mean and standard deviation (SD), or median and interquartile range (IQR), as appropriate. To compare variables among groups, we used Student t test or Mann–Whitney test and one-way ANOVA or Kruskal–Wallis test for numerical variables, and Chi-squared test or Fisher exact test for categorical variables.

To assess the relationship between corticosteroids treatment and endpoints, time-to-event curves were plotted using the Kaplan–Meier method and analysed with Cox regression analysis. For the Kaplan–Meier curves, patients with complementary outcomes were right-censored at the longest recorded LOS. We used inverse probability of treatment weighting (IPW) for baseline differences between treatment groups. We fitted logistic models using the following baseline variables: age, gender, comorbidities (diabetes mellitus and arterial hypertension), APACHE II and SOFA scores, and PaO_2_/FiO_2_ at admission. Weights were calculated following the methodology described elsewhere [11], and a pseudo-population (adjusted sample) was built subsequently. The 95% confidence intervals (CI) were the 2.5th and 97.5th percentiles of the distribution obtained from a nonparametric bootstrap with 1,000 samples. To test the robustness of results, we rerun the primary analysis under several assumptions and scenarios (See Additional file 1).

Furthermore, to assess the relationship between survival and onset of corticosteroids treatment, we fitted a logistic regression where mortality was the dependent variable, and day of starting corticosteroids treatment was the independent variable included as continuous in a linear model, and a restricted cubic splines model to account for potential nonlinear relationships between corticosteroids onset and mortality.

Missing data were not imputed. Analyses were performed in a complete case analysis basis. All tests were two-sided, and a p value < 0.05 was considered statistically significant. Analyses were performed using STATA version 16.

## Results

**Fig. 1:**
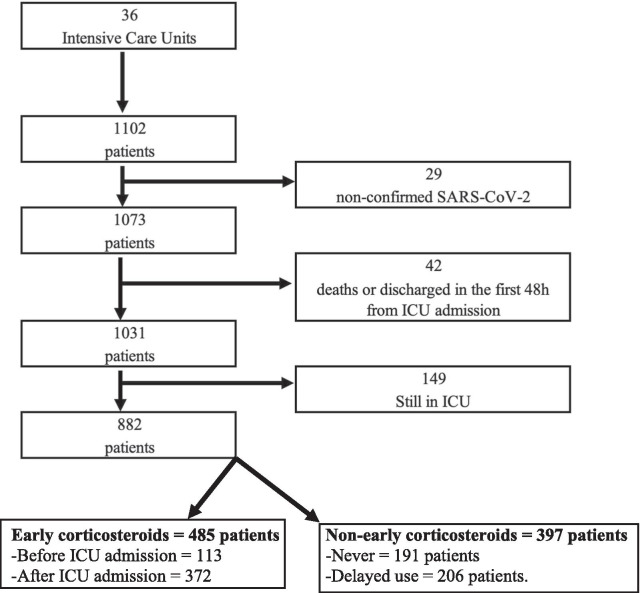
Flow chart of study participants. ICU = Intensive Care Unit

From 1,102 consecutive patients with COVID-19, we analysed 882 patients (Fig. 1). Baseline characteristics are reported in Table 1. Acute hypoxemic respiratory failure was the main reason for ICU admission.

**Table 1:** Baseline characteristics of patients according to the use of corticosteroids. Delayed = after 48 h of ICU admission

	Whole cohort	Never	Early	Delayed	p value
*Patients demographics and comorbidities*					
N (%)	882	191 (21.66%)	485 (54.99%)	206 (23.36%)	
Age, years	62.3 (11.4)	*60.1 (13.9) *^a^	62.9 (10.6)	63.0 (10.8)	0.06
Female (%)	291(33.1%)	66/191 (34.5%)	153/485 (31.5%)	72/204 (35.2%)	0.56
Body mass index (kg/m^2^)	29.2 (5.3)/550	28.71(5.4)/114	29.2(5.3)/292	29.5(5.3)/144	0.39
Arterial hypertension	420/882 (47.6%)	79/191 (41.4%)	240/485 (49.4%)	101/206 (49.0%)	0.14
Diabetes mellitus	201/882 (22.7%)	43/191 (22.5%)	111/485 (22.9%)	47/206 (22.8%)	1.00
Chronic heart failure	13/882 (1.4%)	1/191 (0.5%)	7/485 (1.4%)	5/206 (2.4%)	0.26
Chronic renal failure	52/882 (5.9%)	11/191 (5.7%)	28/485 (5.7%)	13/206 (6.3%)	0.96
Asthma	24/882 (2.7%)	5/191 (2.6%)	16/485 (3.3%)	3/206 (1.4%)	0.42
COPD	40/882 (4.5%)	5/191 (2.6%)	24/485 (4.9%)	11/206 (5.3%)	0.33
Obese	299/792 (37.7%)	66/170 (38.8%)	158/435 (36.3%)	75/187 (40.1%)	0.63
Dyslipidaemia	132/882 (14.9%)	21/191 (11.0%)	76/485 (15.6%)	35/206 (17.0%)	0.18
Cancer	27/882 (3.1%)	3/191 (1.6%)	8/485 (1.6%)	*16/206 (7.8%)*	* < 0.001*
*Laboratory findings*					
Haematocrit (%)	39.2 (5.7)/602	39.1(6.0)/122	39.3(5.6)/313	38.9(5.80)/167	0.62
Platelets, 1000/mm^3^	236 (108)/672	232(106)/130	242(108)/367	222(108)/175	0.11
Leukocytes, 10^3^/μL	9.1(5.8)/663	8.6 (5.2)/129	9.4 (6.1)/360	8.6 (5.5)/174	0.10
Lymphocytes, μL	0.82 (0.72)/658	0.82 (0.45)/126	0.84 (0.87)/361	0.76 (0.49)/171	0.16
CRP, mg/dL	74.5 (98.9)/623	85.4 (110.9)/119	69.0 (93.2)/349	78.5 (101.2)/155	0.08
Lactate, mmol/L	0.43 (0.94)/475	0.39 (0.53)/99	0.44 (0.88)/245	0.46 (1.26)/131	0.27
Ferritin, ng/mL	1665 (1658)/285	1744(2191)/38	1725(1669)/179	1459(1241)/68	0.52
D-Dimer, ng/mL	2026 (2339)/522	*1586 (1957)/96*^a^	2184 (2507)/292	1996 (2177)/134	0.06
CRP/lymphocyte ratio	131 (234)/617	158 (342)/118	110(168)/344	154 (254)/155	0.08
IL-6, pg/mL	253 (486)/87	269 (154)/4	289 (543)/62	143 (308)/21	0.08
LDH, U/L	484 (240)/595	*453 (268)/114*^a^	483 (215)/330	508 (264)/151	0.06
Procalcitonin, ng/mL	1.36 (5.1)/454	0.91 (2.4)/83	1.15 (4.0)/260	2.18 (8.0)/111	0.44
Bilirubin, mg/dL	0.82 (1.16)/567	0.91 (1.46)/98	0.76 (0.61)/322	0.87 (1.72)/147	0.89
AST, U/L	58.3 (86.6)/650	68.5 (148.7)/122	57.2 (60.1)/359	53.3 (72.4)/169	0.45
Creatinine, mg/dL	1.05 (0.68)/658	1.07 (0.76)/124	1.03 (0.66)/361	1.07 (0.66)/173	0.90
Urea, mg/dL	46.5 (28.0)/462	43.7 (35.3)/80	46.6 (27.4)/265	47.9 (23.0)/117	0.05
NTProBNP, pg/mL	1880 (5166)/96	375 (1278)/64	177 (780)/194	89 (451)/83	0.28
*Vital signs and organ support*					
Mechanical ventilation, n (%)	27/882 (3.1%)	2/191 (1.0%)	20/485 (4.1%)	5/206 (2.4%)	0.09
Vasopressor use, n (%)	377/882 (42.7%)	77/191 (40.3%)	214/485 (44.1%)	86/206 (41.8%)	0.64
Renal replacement therapy, n (%)	5/882 (0.6%)	2/191 (1.0%)	2/485 (0.4%)	1/206 (0.5%)	0.62
PaO_2_/FiO_2_	150 (77)/559	153 (78)/107	151 (77)/305	146 (76)/147	0.71
PEEP, cmH2O	13.8 (2.6) / 257	13.5 (2.5) /51	14.0 (2.5) /136	13.4 (2.8) /70	0.15
SpO_2_, %	88.1 (9.2)/641	87.6 (10.6)/136	88.4 (7.8)/342	87.8 (10.4)/163	0.84
Respiratory rate, bpm	25.7 (7.3)/604	26.6 (8.7)/126	*24.8 (6.5)/321*	26.6 (7.3)/157	*0.02*
PaCO2, mmHg	40.2 (12.5) /615	40.4 (13.7) /128	40.0 (11.9) /325	40.5 (12.7) /162	0.91
Temperature, ºC	36.9 (1.1)/654	37.0 (1.1)/137	*36.7 (1.0)/345*	37.1 (1.1)/172	*0.001*
Mean arterial pressure, mmHg	86.7 (15.3)/644	86.9 (14.8)/134	86.8 (15.8)/341	86.3 (14.7)/169	0.96
Heart rate, bpm	85.2 (18.8)/658	88.2 (19.3)/142	84.0 (18.9)/346	84.9 (17.9)/170	0.18
*Severity scores*					
Apache II	13.51 (6.35)/600	12.45 (5.85)/121	13.77 (6.67)/348	13.78 (5.84)/131	0.17
CURB65	1.86 (1.22)/288	1.67 (1.26)/60	1.93 (1.23)/150	1.90 (1.16)/78	0.32
SOFA	5.64 (2.94)/474	5.85 (3.18)/86	5.58 (2.99)/266	5.60 (2.65)/122	0.66

Statistically significant results are in italics

^a^Never significantly different from early and delayed; p = 0.02

COPD: Chronic Obstructive Pulmonary Disease; CRP: C-reactive protein; LDH: lactate dehydrogenase; AST: aspartate aminotransferase; NTProBNP: N-terminal pro-brain natriuretic peptide; APACHE: Acute Physiology and Chronic Health Evaluation; CURB65: confusion, uremia, elevated respiratory rate, hypotension, and aged 65 years or older; SOFA: Sequential Organ Failure Assessment

Four-hundred and eighty-five patients (55.0%) were treated with early corticosteroids (See Additional file 2: Table S1). Corticosteroid exposure did not differ according to age, sex, body-mass index, severity scores, and main comorbidities but small differences were present so that patients receiving early corticosteroids had lower temperature and respiratory rate, and the delayed group had more cancer patients (Table 1). During ICU stay, corticosteroid early treated patients developed less organ dysfunction, had less requirement for renal replacement therapy (RRT), and less systemic inflammation than delayed treated patients (Table 2).

**Table 2:** Evolution of organ failure, vital signs, and laboratory findings during ICU stay according to the use of corticosteroids. Early = in the first 48 h of ICU admission

	Whole cohort	Never	Early	Delayed	p value
n (%)	882	191 (21.7%)	485 (55.0%)	206 (23.3%)	
Mechanical ventilation, n (%)	722/882 (81.9%)	*139/191 (72.8%) *^a^	395/485 (81.4%)	188/206 (91.3%)	* < 0.001*
Vasopressor use, n (%)	639/882 (72.4%)	126/191 (66.0%)	343/485 (70.7%)	*170/206 (82.5%) *^c^	* < 0.001*
Renal replacement therapy, n (%)	96/867 (11.1%)	14/183 (7.6%)	40/479 (8.3%)	*42/205 (20.5%) *^c^	* < 0.001*
SOFA maximum	8.6 (3.7)/729	8.2 (3.5)/132	8.2 (3.5)/426	*9.7 (4.0)/171 *^c^	* < 0.001*
*Vital Signs*					
Temperature maximum, ºC	37.9 (1.0)/869	37.9 (1.0)/183	*37.7 (1.0)/481 *^b^	38.2 (1.0)/205	* < 0.001*
Mean arterial pressure minimum, mmHg	68.8 (13.0)/868	70.7 (13.4)/182	69.1 (13.0)/480	*66.4 (12.3)/206 *^c^	*0.001*
Heart rate maximum, bpm	105.6 (20.9)/868	103.9 (21.7)/182	104.6 (20.9)/480	*109.5 (19.9)/206*^c^	*0.006*
SpO_2_, minimum, %	78.6 (16.2)/869	80.3 (16.2)/184	79.4 (15.1)/480	*75.3 (18.2)/205 *^c^	* < 0.001*
Respiratory rate, maximum, bpm	30 (7.0)/859	31 (7.7)/177	30 (6.6)/477	*32 (7.2)/205 *^c^	*0.005*
Respiratory rate, minimum, bpm	17 (4.4)/859	*19 (5.0)/177 *^a^	17 (4.4)/477	16 (3.8)/205	* < 0.001*
*Arterial blood gas*					
PaO_2_/FiO_2_ minimum	98.3 (47.9)/833	*107.6 (56.9)/167*^a^	96.6 (43.5)/466	94.3 (48.5)/200	*0.03*
PaCO_2_ maximum, mmHg	61.8 (19.3)/855	65.0 (21.2)/178	*58.9 (18.0)/473 *^b^	66.4 (19.3)/204	* < 0.001*
*Laboratory findings*					
Ferritin maximum, ng/mL	2305 (2257)/667	*1771 (1863)/104*^a^	2314 (2251)/389	2604 (2432)/174	*0.003*
D-Dimer maximum, ng/mL	5124 (2983)/809	*4124 (2823)/158 *^a^	5176 (3008)/452	5799 (2848)/199	* < 0.001*
CRP maximum, mg/dL	121 (138)/845	124 (140)/172	111 (128)/472	*142 (154)/201 *^c^	*0.005*
Lymphocytes minimum, mL	0.45 (0.34)/848	*0.60 (0.41)/175 *^a^	0.42 (0.31)/471	0.39 (0.29)/202	* < 0.001*
CRP/ lymphocyte ratio maximum	273 (447)/843	237 (382)/171	245 (389)/471	*368 (590)/201 *^c^	*0.004*
IL-6 maximum, pg/mL	957 (1787)/314	545 (1142)/22	933 (1803)/218	1148 (1884)/74	0.14
LDH maximum, U/L	648 (382)/828	*566 (339)/162 *^a^	659 (394)/467	686 (379)/199	* < 0.001*
Leukocytes maximum, 10^3^/mL	15.5 (10.0)/843	*13.1 (7.7)/173 *^a^	16.2 (10.5)/467	15.9 (10.3)/203	*0.001*
Procalcitonin maximum, ng/mL	4.1 (9.9)/756	3.4 (9.5)/143	3.1 (7.3)/434	*7.1 (14.2)/179 *^c^	* < 0.001*
Platelets maximum, 1000/mm^3^	387 (153)/852	390 (152)/176	377 (149)/473	*405 (161)/203 *^c^	*0.03*
Bilirubin, maximum, mg/dL	2.3 (3.7)/803	*1.8 (2.2)/152 *^a^	2.3 (3.4)/461	2.9 (4.8)/190	*0.001*
AST maximum, U/L	198 (372)/845	*145 (274)/175 *^a^	199 (346)/470	240 (483)/200	* < 0.001*
Creatinine maximum, mg/dL	1.8 (1.7)/851	1.8 (1.8)/175	1.7 (1.5)/473	*2.2 (1.9)/203 *^c^	*0.02*
Urea maximum, mg/dL	115 (161)/764	117 (316)/157	106 (69)/423	*135 (106)/184 *^c^	* < 0.001*
NTProBNP, maximum pg/mL	1996 (4815)/261	2573 (6712)/41	*1684 (4693)/152 *^b^	2344 (3590)/68	*0.04*
Haematocrit minimum, %	38.2 (6.0)/834	37.0 (6.4)/171	*38.8 (5.8)/464 *^b^	37.8 (5.7)/199	*0.003*
Lactate maximum, mmol/L	0.4 (0.8)/692	0.3 (0.5)/142	0.4 (0.7)/392	0.4 (1.2)/158	0.33

Statistically significant results are in italics

^a^Never significantly different from early and delayed

^b^early significantly different from never and delayed

^c^delayed significantly different from never and early

SOFA: Sequential Organ Failure Assessment; CRP: C-reactive protein; LDH: lactate dehydrogenase; AST: aspartate Aminotransferase; NTProBNP: N-terminal pro-brain natriuretic peptide

### Primary outcome

**Fig. 2:**
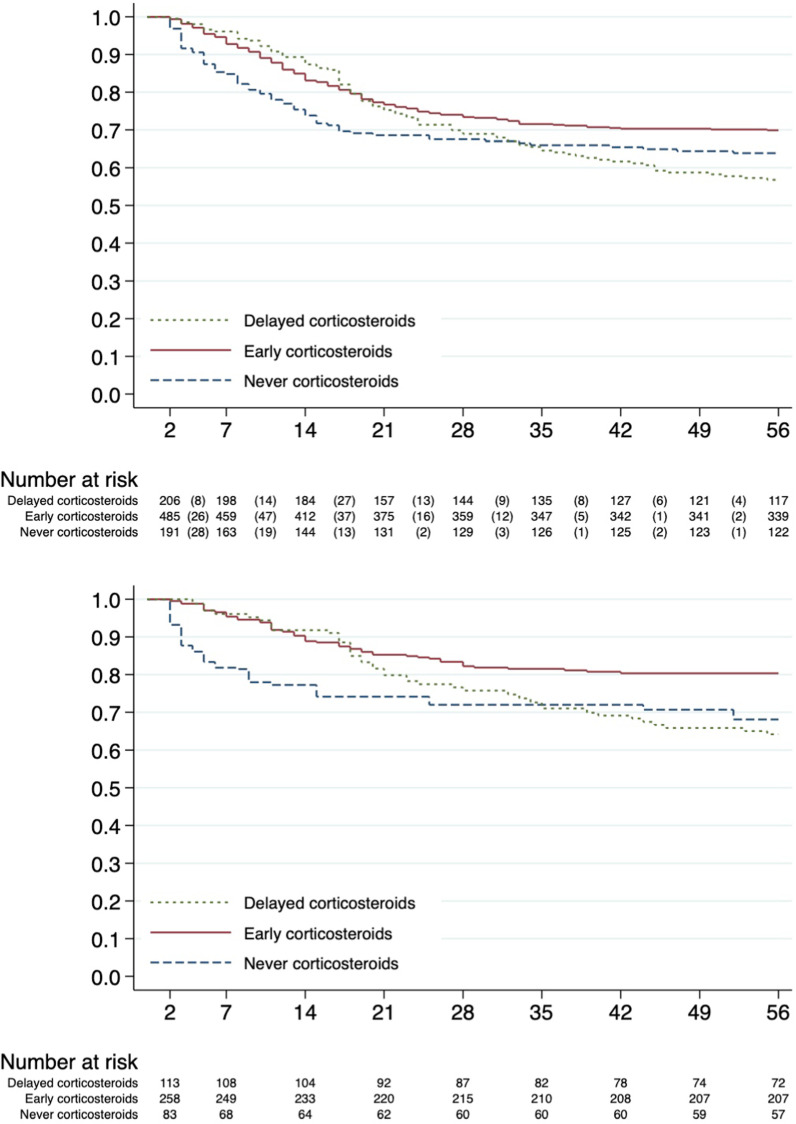
Kaplan–Meier estimates of mortality according to the use of corticosteroids. Delayed = after 48 h of ICU admission. The upper graphs are crude estimates. The lower graphs are inverse probability weighted (IPW) estimates (N = 455)

Overall ICU mortality was 34.9% (n = 308), significantly lower in the early corticosteroids group (30.3%) than in non-early treated group (40.3%) (HR 0.71, 95% CI 0.57–0.89) (Table 3, Fig. 2). A sensitivity analysis showed less mortality reduction with corticosteroids in women, patients < 60-year-old, with hypertension, cancer, or type 2 diabetes, CRP < 10 mg/dL, D-Dimer > 1500 ng/mL, ICU admission > 8 days after the onset of symptoms, corticosteroids within 7 days of symptom onset, Pa/FiO_2_ > 200, or APACHE score > 14 at ICU admission (Additional file 3: Table S2). The reduction in mortality with corticosteroids was independent of treatment with tocilizumab, initiation of steroids before ICU admission, need of invasive mechanical ventilation (MV), lymphocytes count, duration of corticosteroids treatment, or the admitting hospital (Additional file 3: Table S2). This reduction in mortality was also observed when including the 149 patients not discharged from ICU or when including “missing” as a category in the IPW cohort .

**Table 3:** Outcomes according to early corticosteroids use in the first 48 h of ICU admission, compared with never or delayed use

	Whole cohort	Never	Early	p value	Delayed	p value
n (%) n IP-weighted (%)	882 455	191 (21.7%) 84 (18.5%)	485 (55.0%) 259 (57.0%)		206 (23.4%) 112 (24.6%)	
ICU mortality, cases/person-days	308 (34.9%) / 57,588	70 (36.6%) /11,893	147 (30.3%) /33,147		91 (44.2%) /12,547	
ICU mortality (hazard ratio)		1 (Ref.)	0.75 (0.56–1.00)	0.05	1.10 (0.81–1.50)	0.54
ICU mortality (cases/person-days; hazard ratio) IP-weighted	116 (25.5%) / 33,198	21 (25%) / 5982 1 (Ref.)	52 (20.1%) / 19,821 0.55 (0.31. 0.95)	*0.02*	43 (38.4%) / 7394 1.06 (0.61–1.85)	0.84
7-day mortality, cases/person-days	72 (8.2%) /4229	29 (15.2%) /864	35 (7.2%) /2355		8 (3.9%) /1009	
7-day mortality (hazard ratio)		1 (Ref.)	0.45 (0.27–0.73)	*0.001*	0.24 (0.11–0.52)	* < 0.001*
7-day mortality (cases/person-days; hazard ratio) IP-weighted	27 (6.0%) / 2205	12 (14.3%) / 377 1 (Ref.)	11 (4.2%) / 1276 0.23 (0.10–0.55)	* < 0.001*	4 (3.6%) / 553 0.20 (0.06–0.62)	*0.006*
ICU length of stay, days	17.8 (14.2)	14.2 (13.0)	16.4 (12.5)		24.7 (16.7)	
ICU length of stay (mean difference)		0 (Ref.)	2.2 (− 0.1 to 4.5)	0.06	10.6 (7.9–13.3)	* < 0.001*
ICU length of stay (days; mean difference) IP-weighted	18.1 (15.0)	13.0 (12.7) 0 (Ref.)	16.1 (12.3) 2.6 (− 1.0 to 6.3)	0.16	26.6 (18.5) 13.0 (8.2. 17.9)	* < 0.001*
ICU length of stay among survivors, days	18.0 (14.7)	15.0 (13.0)	16.6 (13.1)		25.7 (18.1)	
ICU length of stay among survivors (mean difference)		0 (Ref.)	1.6 (−1.4 to 4.6)	0.29	10.7 (7.1–14.4)	* < 0.001*
ICU length of stay among survivors (days; mean difference) IP-weighted	17.7 (15.0)	13.7 (12.4) 0 (Ref.)	15.9 (12.6) 2.0 (−1.7 to 5.7)	0.29	26.7 (19.7) 12.9 (7.1–18.7)	* < 0.001*
Ventilatory-free days	8.4 (9.4)	9.6 (10.1)	9.6 (9.5)		4.7 (7.3)	
Ventilatory-free days (mean difference)		0 (Ref.)	− 0.00 (− 1.5 to 1.5)	0.99	− 4.9 (− 6.70 to − 3.09)	* < 0.001*
Ventilatory-free days (days; mean difference) IP-weighted	9.9 (9.4)	12.3 (9.9) 0 (Ref.)	11.3 (9.3) 0.23 (− 2.41 to 2.87)	0.86	5.0 (7.4) − 5.9 (− 8.7 to − 3.5)	* < 0.001*
Medical complications, n (%)	860 (97.5%)	179 (93.7%)	477 (98.3%)		204 (99.0%)	
Medical complications (odds ratio)		1 (Ref.)	4.00 (1.61–9.94)	*0.003*	6.84 (1.51–30.96)	*0.01*
Medical complications (n %; odds ratio) IP-weighted	443 (97.4%)	78 (92.9%) 1 (Ref.)	255 (98.5%) 3.72 (1.0–13.87)	0.05	110 (98.21%) 2.95 (0.57–15.32)	0.20
Infectious complications, n (%)	509 (57.7%)	92 (48.2%)	273 (56.3%)		144 (69.9%)	
Infectious complications (odds ratio)		1 (Ref.)	1.39 (0.99–1.94)	0.05	2.50 (1.66–3.77)	* < 0.001*
Infectious complications (n %; odds ratio) IP-weighted	259 (56.9%)	35 (41.7%) 1 (Ref.)	139 (53.7%) 1.54 (0.89–2.66)	0.12	85 (75.9%) 4.29 (2.23–8.25)	* < 0.001*

Statistically significant results are in italics

We found a statistically significant linear association between the day of starting corticosteroids treatment and mortality (Additional file 4: Figure S1). However, patients who started corticosteroids before ICU admission showed a higher mortality rate (crude mortality 39.8%) as compared to those who started corticosteroids after ICU admission (crude mortality 27.4%). This difference disappeared in the adjusted model (p = 0.40) (Additional file 1: Figure S2), pointing to a selection bias—those who failed to respond to corticosteroids were admitted to ICU—that precludes further conclusions about pre-ICU corticosteroids treatment. These results indicate that the earlier the better, after ICU admission.

ICU mortality was lower when using moderate-to-high doses of corticosteroids (26.9% vs. 32.8% with low dose (HR 0.58, 95% CI 0.45–0.75) (Additional file 1: Table S3–S4, Additional file 1: Figure S3).

### Secondary outcomes

Patients treated with early corticosteroids had shorter ICU LOS, more ventilatory-free days, lower rate of acute renal failure, less need of vasopressors, fewer infections and less inflammation and organ failure than delayed treated patients. There were no differences in the rate of medical complications between groups (Tables 2–3).

Regarding corticosteroids **doses**, early use of moderate-to-high doses, compared with low doses, was associated with a shorter ICU LOS, lower organ dysfunction, less requirement of MV or RRT, and no increase in medical or infectious complications (Additional file 1: Table S3–S4).

**Fig. 3:**
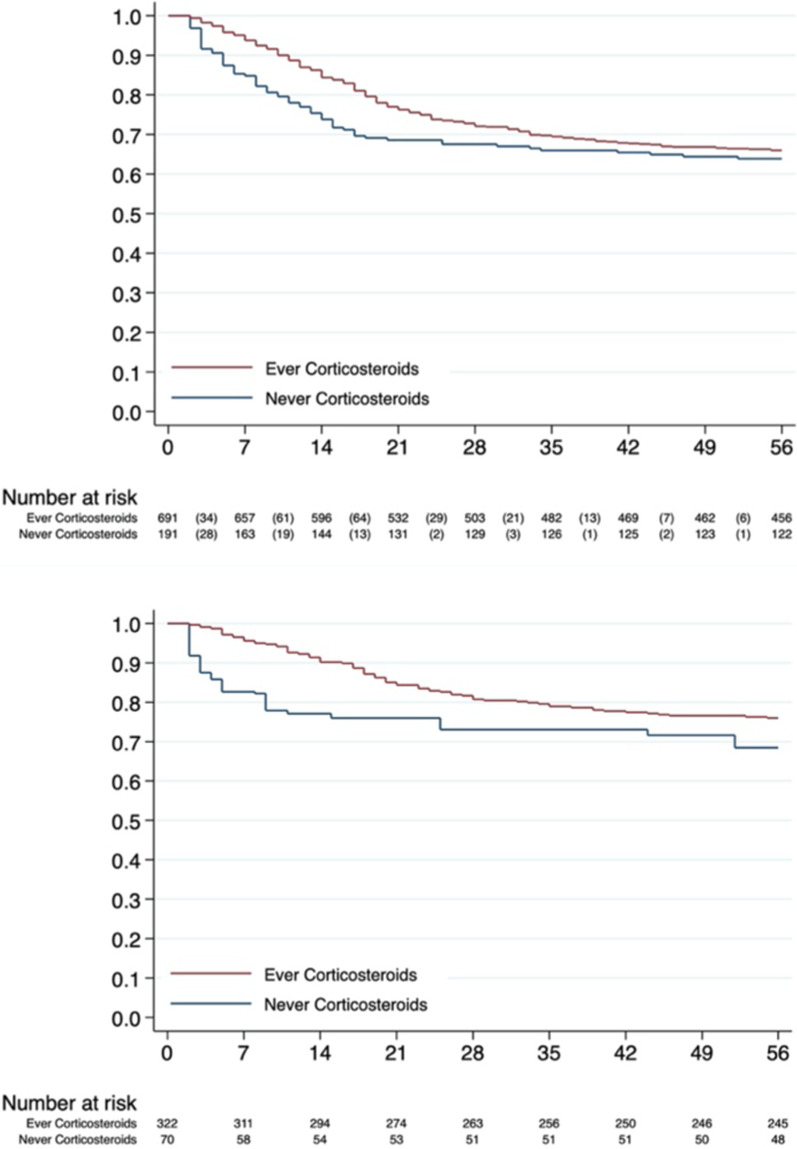
Kaplan–Meier estimates of mortality according to corticosteroids use during total hospital stay: ever (early + delayed) or never treatment. The upper graphs are the crude estimates. The lower graphs are IP-weighted estimates (N = 392)

Patients who **never** received corticosteroids were a less severe population: younger, with fewer signs of organ damage and inflammation, and lower requirement for MV. Nonetheless, compared with early treated patients, they had higher ICU mortality (36.6% vs. 30.3%, HR 0.55, 95% CI 0.35–0.93) and higher early 7-days mortality (Table 3, Additional file 1: Figure S4). In contrast, compared with ever-treated patients, they had no difference in ICU survival, but lower LOS, less requirement for mechanical ventilation and vasopressors, better disease progression, and a lower number of infectious complications (Additional file 1: Tables S5-S6, Fig. 3).

## Discussion

The major findings of our study are that early use of corticosteroids in critically ill patients with COVID-19 was associated with: (i) lower ICU mortality when compared to delayed or no use of corticosteroids; (ii) shorter ICU LOS; (iii) decreased organ dysfunction, and (iv) fewer days on MV, with no increase in medical or infectious complications. These findings remained statistically significant after adjusting for age, gender, comorbidities, severity, and PaO_2_/FiO_2_ at admission.

To study the influence of corticosteroids on mortality in our cohort, we set a cut-off point of 48 h after admission to ICU based on the comparison of survivors vs. non-survivors where we found that the onset time for using corticosteroids was clinically relevant. Furthermore, we consider this period long enough for an experienced clinician to assess patients’ response to initial support and therapy and modify them accordingly. We have tried to solve the question placed by many clinicians: shall I start corticosteroids in this patient? Like an intention-to-treat analysis, patients receiving corticosteroids after 48 h of ICU admission were not excluded because the decision to start therapy in this group was probably guided by other uncontrolled factors.

Several recent publications support the early and selective use of corticosteroids in symptomatic patients infected with SARS-COV-2. The RECOVERY trial found a reduction in 28-day mortality in hospitalized COVID-19 patients treated with dexamethasone if they required oxygen or MV [4]. The prospective meta-analysis of 7 randomized trials performed by the WHO Rapid Evidence Appraisal for COVID-19 Therapies Working Group found that 28-day all-cause mortality in critically ill patients with COVID-19 was lower among those who received corticosteroids compared with those who received usual care or placebo (summary odds ratio, 0.66) [5]. Our study reinforces those results and sheds more light by providing new and more complete information. Of note, these findings are in contrast with previous reports on corticosteroid therapy in past outbreaks of other coronaviruses (SARS-CoV, MERS-CoV), or other viral pneumonia (Influenza, Respiratory Syncytial Virus) [12–16].

Our sensitivity analysis showed that early use of corticosteroids was not as effective in women, in those with lower risk of death –younger patients with good oxygenation and less inflammation—and neither in those with greater risk or severity –cancer, diabetics, D-Dimer > 1500 ng/mL, APACHE score > 14—. These findings suggest that patient characteristics should be assessed before prescribing corticosteroids.

In clinical practice, corticosteroids are used in most critically ill patients [16]. The extensive use of corticosteroids in our cohort reflects the severity of our patients (almost 60% directly admitted to ICU upon arrival to the hospital, 81.9% with invasive mechanical ventilation in the first 48 h after ICU admission, and with a mean PaO2/FiO2 = 150). Since observational studies are prone to selection bias, we used inverse probability of treatment weighting to tackle this problem. Corticosteroid therapy is further entangled by other factors that merit discussion, including timing, type of corticosteroids, duration of treatment, and dosing.

Our average time to corticosteroids administration was 12 days after symptom onset (Additional file 2: Table S1), like the 13 days in the RECOVERY study for mechanically ventilated patients [4]. The decision to initiate corticosteroid therapy in our patients was guided when signs of hyperinflammation and severity of respiratory failure were evident. The delay or non-use of corticosteroids in 45% of our patients may reflect the controversy on their benefit/harm profile, WHO recommendations [1], or significant changes in clinical evolution. Ideally, we should start corticosteroid therapy in the initial phases of the hyperinflammatory state. Early use in the absence of hyperinflammation could be harmful, especially in the initial stage of viral replication [4, 17, 18]. Similar to the RECOVERY trial, we have also found that corticosteroids were not associated with a reduction in mortality among those patients with symptoms duration under 7 days. One possible explanation is that corticosteroids may slow viral clearance in such an early phase. However, studies on viral clearance have yielded contradictory results [19–22]. Unfortunately, we did not collect time to viral clearance.

Interestingly, patients who never received corticosteroids had higher mortality than early treated patients, a difference that was not observed when we compare them with ever-treated patients, that includes early plus delayed treatment. Delayed corticosteroids, when advanced organ damage already exists, might be ineffective and even detrimental, as previously described in ARDS [23], with increased infectious complications and mortality, counteracting the positive effect of early treatment. The absence of benefit with too early use of corticosteroids (within 7 days of symptom onset), together with the beneficial effect of early use, and the worst results with delayed use, reveals a U-shaped time-outcome relationship. Although we do not know the optimal time to start corticosteroids, probably patients with elevated inflammatory markers after seven days of symptoms, requiring oxygen or ventilatory support may benefit the most, whereas those who have not received corticosteroids in early phases, probably will not benefit afterwards.

To the best of our knowledge, recommendations about dose and duration of corticosteroid treatment are empiric. With large doses and long treatments, the potential for adverse effects increases, and the possible benefit is lost [24]. The Italian National Institute for Infectious Diseases recommends a 10-day regimen: 5-days full dose of methylprednisolone 1 mg/Kg daily or dexamethasone 20 mg daily, and five days for tapering [25]. In general, most regimens for acute hyperinflammatory states recommend treatments shorter than two weeks [25, 26]. Most common dose regimens range between 0.5–2 mg/kg/day of methylprednisolone, equivalent to 0.1–0.4 mg/kg/day of dexamethasone, defined in our study as moderate-to-high doses. The cut-off points were defined before the publication of the RECOVERY trial that used a fixed dexamethasone dose (6 mg) lower than described in the literature for ARDS [10]. We observed a greater mortality reduction with a moderate-to-high dose regimen, similar to other studies [6, 9, 27]. Although our classification is artificial, it favours a higher dose of corticosteroids. As a result of the RECOVERY trial, the NIH and the WHO recommend a low fixed dose of daily dexamethasone [7, 8], and numerous studies with different corticosteroids and doses were prematurely stopped without completion [5, 9], but future comparative studies with higher doses are warranted [6, 10].

Also, the beneficial effects of early corticosteroids in our patients expand beyond an absolute reduction in all-cause mortality, including a shorter ICU LOS, less organ dysfunction, and an increase in ventilator-free days.

This study has several strengths. First, this multicentre nationwide prospective data collection with over 1,000 patients from 36 ICUs provides a very detailed description of all gathered data from ICU admission to death or ICU discharge. Second, to the best of our knowledge, this is the first observational study that prospectively explores the association between different doses and timings of corticosteroid therapy in COVID-19 patients and ICU mortality. Third, we have used IPW to control for confounding with pre-specified demographic, comorbidities, and severity parameters. However, we acknowledge some limitations of our study. First, the observational nature of our study design, which may be subject to biases. Although we adjusted for likely confounders, some unmeasured confounding is still possible. Second, we cannot exclude missing data for some variables and potential for inaccuracies in the electronic health records due to the burden of care experienced by participating clinicians during the pandemic. However, due to the nature of our registry, we consider that selection bias was not favoured, and our analyses are valid. Third, although moderate-to-high doses of dexamethasone were most effective, no firm conclusions can be drawn on the drug or the dose, as our hypothesis and the definition of variables in the protocol preclude to do so, and doses predefined as low are not completely equivalent and could be controversial. Finally, at the time of the analysis, 149 (13.5%) patients did not have a definitive outcome regarding status at ICU discharge and were not included in the main analysis, although included in the sensitivity analyses.

## Conclusions

In conclusion, in critically ill COVID-19 patients with acute respiratory failure, the use of corticosteroids within the first 48 h of ICU admission was associated with a marked reduction in ICU mortality and ICU LOS. We also found a clear relationship between exposure and a beneficial effect on organ dysfunction. Further research is needed to characterize the optimal drug, onset, dose, and duration of corticosteroids therapy in this patient population.

### Supplementary information

**Additional file 1**. Methods, Tables S3–6, Figures S2-5 and References.

**Additional file 2**. ** Table S1**: Use of corticosteroids and combination of treatments.

**Additional file 3**. ** Table S2**: Sensitivity analyses: ICU mortality comparing early versus non-earlyuse of corticosteroids.

**Additional file 4**. ** Figure S1**: Restricted cubic spline to plot the odds ratio (95% confidenceinterval) of mortality according to the onset day of corticosteroids treatment.

## Data Availability

After publication, data will be made available to other investigators on reasonable requests to the corresponding author. A proposal with a detailed description of study objectives and statistical analysis plan will be needed for evaluation of the reasonability of requests. Additional materials might also be required during the process of evaluation. Deidentified participant data will be provided after approval from the corresponding author.

## Abbreviations

APACHE: Acute Physiology and Chronic Health disease Classification System
ARDS: Acute respiratory distress syndrome
CI: Confidence interval
COVID-19: Coronavirus disease 2019
CRP: C-reactive protein
HR: Hazard ratio
ICU: Intensive Care Unit
IPW: Inverse probability of treatment weighting
IQR: Interquartile range
LOS: Length of stay
MV: Mechanical ventilation
NIH: US National Institutes of Health
RRT: Renal replacement therapy
SARS-CoV-2: Severe acute respiratory syndrome coronavirus 2
SD: Standard deviation
SOFA: Sequential Organ Failure Assessment
WHO: World Health Organization
