# Publisher Correction: Early corticosteroids are associated with lower mortality in critically ill patients with COVID‑19: a cohort study

**DOI:** 10.1186/s13054-023-04711-3

**Published:** 2023-12-11

**Authors:** Pablo Monedero, Alfredo Gea, Pedro Castro, Angel M. Candela‑Toha, Maria L. Hernandez‑Sanz, Egoitz Arruti, Jesus Villar, Carlos Ferrando, COVID-19 Spanish ICU Network, Pablo Monedero, Alfredo Gea, Pedro Castro, Angel M. Candela-Toha, Marina Vendrell, Gerard Sánchez-Etayo, Amalia Alcón, Isabel Belda, Mercé Agustí, Albert Carramiñana, Isabel Gracia, Miriam Panzeri, Irene León, Jaume Balust, Ricard Navarro, María José Arguís, María José Carretero, Cristina Ibáñez, Juan Perdomo, Antonio López, Manuel López-Baamonde, Tomás Cuñat, Marta Ubré, Antonio Ojeda, Andrea Calvo, Eva Rivas, Paola Hurtado, Roger Pujol, Nuria Martín, Javier Tercero, Pepe Sanahuja, Marta Magaldi, Miquel Coca, Elena del Rio, Julia Martínez-Ocon, Paula Masgoret, Monserrat Tio, Angel Caballero, Raquel Risco, Raquel Bergé, Lidia Gómez, Nicolás de Riva, Ana Ruiz, Beatriz, Tena, Sebastián Jaramillo, José María Balibrea, Francisco Borja de Lacy, Ana Otero, Ainitze Ibarzabal, Raquel Bravo, Anna Carreras, Daniel Martín-Barreda, Alfonso Jesús Alias, Mariano Balaguer, Jorge Aliaga, Alex Almuedo, Joan Ramón Alonso, Rut Andrea, Gerard Sergi Angelès, Marilyn Arias, Fátima Aziz, Joan Ramon Badía, Enric Barbeta, Toni Torres, Guillem Batiste, Pau Benet, Xavi Borrat, María Borrell, Ernest Bragulat, Inmaculada Carmona, Manuel Castellà, Pedro Castro, Joan Ceravalls, Oscar Comino, Claudia Cucciniello, Clàudia De Deray, Oriol De Diego, Paula De la Matta, Marta Farrero, Javier Fernández, Sara Fernández, Anna Fernández, Miquel Ferrer, Ana Fervienza, María Tallo Forga, Daniel Forné, Clàudia Galán, Andrea Gómez, Eduard Guasch, María Hernández-Tejero, Adriana Jacas, Beltrán Jiménez, Pere Leyes, Teresa López, José Antonio Martínez, Graciela Martínez-Pallí, Jordi Mercadal, Guido Muñoz, José Muñoz, Ricard Navarro, Josep María Nicolás, José Tomás Ortiz, Anna Peiró, Manuel Pérez, Esteban Poch, Margarida Pujol, Eduard Quintana, Bartomeu Ramis, Enric Reverter, Irene Rovira, Pablo Ruiz, Elena Sandoval, Stefan Schneider, Oriol Sibila, Carla Solé, Alex Soriano, Dolors Soy, M. Suárez, Adrián Téllez, Néstor David Toapanta, Antoni Torres, Xavier Urra, César Aldecoa, Alicia Bordell, Silvia Martín, Judith Andrés, Alberto Martínez Ruiz, Gonzalo Tamayo Medel, Iñaki Bilbao Villasante, Fernando Iturri Clavero, Covadonga Peralta Álvarez, Julia T. Herrera Díez, Andrea García Trancho, Iñaki Sainz Mandiola, Carmen Ruano Suarez, Angela Ruiz Bocos, Eneritz Urrutia Izagirre, Pablo Ortiz de Urbina Fernández, Naiara Apodaka López, Leire Prieto Molano, Eunate Ganuza Martínez, Iratxe Vallinas Hidalgo, Karmele de Orte Sancho, Celia González Paniagua, Gemma Ortiz Labrador, Mireia Pérez Larrañaga, Marta López Miguelez, Estíbaliz Bárcena Andrés, Erik Urutxurtu Laureano, Maria Jesús Maroño Boedo, Blanca Escontrela Rodríguez, Aitziber Ereñozaga Camiruaga, Deiene Lasuen Aguirre, Ainhoa Zabal Maeztu, Ane Guereca Gala, Iker Castelo Korro, Andrés Álvarez Campo, Alejandro Carcelen Viana, Alejandro Alberdi Enríquez, Xabier Ormazábal Rementeria, Alberto Sánchez Campos, Rosa Gutiérrez Rico, Pablo Barbier Damborenea, Marta Guerenabarrena Momeñe, Borja Cuesta Ruiz, Alejandro López Rico, Ana Rojo Polo, Covadonga García Grijelmo, Mikel Celorrio Reta, Eneko Martín Arroyo, Leire Artaza Aparicio, Iñaki Ituarte Aspiazu, Ane Igeregi Basabe, Itxaso Merino Julian, Isabel Diaz Rico, Maria Paz Martínez, Ramón Adalia Bartolomé, Luigi Zattera, Irina Adalid Hernandez, Leire Larrañaga Altuna, Aina Serrallonga Castells, Adriana Vílchez Garcia, María Núñez, Lorena Román, Isabel Ramos Delgado, Adela Benítez-Cano Martínez, Mireia Chanzá Albert, Juan Carlos Álvarez García, Luis Aguilera Cuchillo, Sandra Beltrán de Heredia, Jesús Carazo Cordobés, Carlos Alberto García Bernedo, Fernando Escolano Villén, Francisco Javier Redondo Calvo, Rubén Villazala González, Victor Baladron González, Patricia Faba, Omar Montenegro, Natalia Bejarano Ramírez, Sergio Marcos Contreras, Alejandro Garcia Rodríguez, Saleta Rey Vázquez, Cristina Garcia Pérez, Eva Higuera Miguelez, Irene Pérez Blanco, David García Rivera, Ane Martín de la Fuente, Marta Pardo, Vanessa Rodriguez, Unai Bengoetxea, Fernando Ramasco, Sheila Olga Santidrián Bernal, Alvar Santa Cruz Hernando, Antonio Planas Roca, Carlos Figueroa Yusta, Esther García Villabona, Carmen Vallejo Lantero, Eva Patiño Rodriguez, Alvaro Esquivel Toledo, David Arribas Méndez, Mar Orts Rodriguez, Rosa Méndez Hernández, Jesús Nieves Alonso, Inés Imaz Artazcoz, Sonia Expósito Carazo, Carlos Román Guerrero, Elena Rojo Rodríguez, Ricardo Moreno González, Julia Hernando Santos, Jara Torrente Pérez, Esperanza Mata Mena, Manuel José Muñoz Martínez, Enrique Alday Muñoz, Patricia Martin Serrano, Laura Cotter Muñoz, Amadea Mjertan, Diego Gutierrez Martínez, Carmen Rodríguez García, Olaya Alonso Viejo, Juan Alvarez Pereira, Ana Carmona Bonet, Diana Parrado López, Eva de Dios Tomas, Rafael Martín Celemin, María Luisa Meilan Paz, Luis Quecedo Gutiérrez, Noemí Diaz Velasco, Gabriel Martin Hernández, Francisco Garcia del Corral, Gloria Hernandez Arias, David Rodriguez Cuesta, Ana Gómez Rice, Encarna Mateos Sevillano, Natalia Olmos Molpeceres, Beatriz Domínguez, Ana Vázquez Lima, Ángel Candela, Ismael AAcevedo Bambaren, Maria Isabel Albala Blanco, Paloma Alonso Montoiro, Fernando Álvarez Utrera, Juan Avellanosa Esteruelas, Amal Azzam López, Alberto José Balvis Balvis, Tommaso Bardi, María Beltrán Martín, Jacobo Benatar Haserfaty, Alberto Berruezo Camacho, Laura Betolaza Weimer, María del Mar Carbonell Soto, Cristina Carrasco Seral, Cristina Cerro Zaballos, Elizabeth Claros Llamas, Pilar Coleta Orduna, Ingrid P. Cortes Forero, Pascual Agustín Crespo Aliseda, María Angélica de Pablo Pajares, Yolanda Díez Remesal, Trinidad Dorado Díaz, Noemí Echevarría Blasco, María Elena Elías Martín, Javier Felices Triviño, Natalia Fernández López, Cristina Fernández Martín, Natalia Ferreiro Pozuelo, Luis Gajate Martín, Clara Gallego Santos, Diego Gil Mayo, María Gómez Rojo, Claudia González Cibrián, Elena Herrera López, Borja Hinojal Olmedillo, Berta Iglesias Gallego, Sassan Khonsari, María Nuria Mane Ruiz, María Manzanero Arroyo, Ana María Mariscal Ortega, Sara Martín Burcio, María del Carmen Martín González, Ascensión Martín Grande, Jose Juan Martín López, Cecilia Martín Rabes, Marcos Martínez Borja, Nilda Martínez Castro, Adolfo Martínez Pérez, Snejana Matcan, Cristina Medrano Viñas, Lisset Miguel Herrera, Adrián Mira Betancur, María Montiel Carbajo, Javier Moya Moradas, Lorena Muñoz Pérez, Mónica Nuñez Murias, Eva Ordiales González, Óscar Ordoñez Recio, Miguel Ángel Palomero Rodriguez, Diego Parise Roux, Lucia Pereira Torres, David Pestaña Lagunas, Juana María Pinto Corraliza, Marian Prieto Rodrigo, Inmaculada Rodriguez Diaz-Regaño, David Rodriguez Esteban, Víctor Rojas Pernia, Álvaro Ruigómez Saiz, Bárbara Saavedra Villarino, Noemí Samaranch Palero, Gloria Santos Pérez, Jaume Serna Pérez, Ana Belén Serrano Romero, Jesús Tercero López, Carlos Tiscar García, Marta de la Torre Concostrina, Eva María Ureta Mesa, Eva Velasco Olarte, Judith Villahoz Martínez, Raúl Villalaba Palacios, Gema Villanueva García, Cristina Vogel de Medeiros, Soraya Gholamian Ovejero, Marta Vicente Orgaz, Patricia Lloreda Herradon, Cristina Crespo Gómez, Tatiana Sarmiento-Trujillo, Noemí García Medina, María Martínez García, Carles Espinós Ramírez, Nabil Mouhaffel Rivero, Jose Antonio Bernia Gil, Sonsoles Martín, María Victoria Moral, Josefina Galán, Pilar Paniagua, Sergio Pérez, Albert Bainac, Ana Arias, Elsa Ramil, Jorge Escudero, Pablo Monedero, Carmen Cara, Andrea Lara, Elena Mendez Martínez, Jorge Mendoza, Íñigo Rubio Baines, Carmen Sala Trull, Pablo Montero López, Alfredo Gea, Alejandro Montero, Rocío Armero Ibañez, Juan Vicente Llau Pitarch, Fernando Rauer Alcóver, Cristina Álvarez Herreros, Cyntia Sánchez Martín, Lucía López Ocáriz Olmos, Marta Navas Moruno, Fernando García Montoto, M. F. Mirón Rodriguez, Laura Fuentes Coco, Cristina Hernández Gamito, Antonio Barba Orejudo, Luis Gerardo Smith Vielma, Yasmina González Marín, Francisco de Borja Amador Penco, Marta Donoso Domínguez, Silvia Esquivel Ramírez, José Antonio Carbonell, Berta Monleón López, Sara Martínez-Castro, Gerardo Aguilar, María Gestal, Pablo Casas, Angel Outeiro Rosato, Andrea Naveiro Pan, María Alonso Portela, Adrián García Romar, Eva Mosquera Rodríguez, Diego Ruanova Seijo, Pablo Rama Maceiras, Francisco Castro-Ceoane, Esther Moreno López, Sergio Gil, Julia Guillén Antón, Patricia García-Consuegra Tirado, Aurora Callau Calvo, Laura Forés Lisbona, María Carbonell Romero, Belén Albericio Gil, Laura Pradal Jarne, María Soria Lozano, Diego Loscos López, Andrea Patiño Abarca, Javier Pérez-Asenjo, Ángel Díez-Domínguez, Ion Zubizarreta, Jon Ramos, Iosu Fernández, Emilio Maseda, Alejandro Suárez de la Rica, Javier Veganzones, Itziar Insausti, Javier Sagra, Sofía Díaz Carrasco, Ana Montero Feijoo, Julio Yagüe, Ignacio Garutti, Javier Hortal, Patricia Piñeiro, Matilde Piñeiro, Matilde Zaballos, Jamil cedeño, Pablo García-Olivares, Alberto Garriido, Jose Eugenioi Guerrero, Eva Bassas Parga, Carmen Deiros Garcia, Elisenda Pujol Rosa, Ana Tejedor Navarro, Roser Font Gabernet, Maria José Bernat, Meritxell Serra Valls, Cristina Cobaleda Garcia-Bernalt, Jesus Fernanz Anton, Adriana Aponte Sierra, Lucia Gil Gomez, Olaia Guenaga Vaqueiro, Susana Hernandez Marin, Laura Pardo Pinzon, Sira Garcia Aranda, Carlos Briones Orejuela, Edgar Cortes Sánchez, Alejandro Romero Fernández, Esther Fernández SanJosé, Patricia Iglesias Garsabal, Guillermo Isidro Lopez, Ana Vicol, Sara Espejo Malagon, María Sanabra Loewe, Laura Grau Torradeflo, Lourdes Blanco Alcaide, Gloria Buenaventura Sanclemente, Pere Serra Pujol, Gustavo Cuadros Mendoza, Miroslawa Konarska, Fedra Bachs Almenara, Agnieszka Golska, Aleix Carmona Blesa, Arantxa Mas Serra, Javier Ripolles Melchor, Ana Nieto Moreno, Káteri Chao Novo, Sandra Gadín López, Elena Nieto Moreno, Bérénice Gutiérrez Tonal, Elena Lucena de Pablo, Barbara Algar Yañez, Beatriz Vázquez Rivero, Beatriz Nozal Mateo, Marina de Retes, Norma Aracil Escoda, Cristina Gallardo Mayo, Rosa Sanz González, Alicia Ruiz Escobar, Maria Laura Pelegrina López, Marina Valenzuela Peña, David Stolle Dueñas, Ane Abad Motos, Alfredo Abad-Gurumeta, Ana Tirado Errazquin, Elena Sáez Ruiz, Nerea Gómez Pérez, Francisco de Borja Bau González, Cesar Morcillo Serra, Jessica Souto Higueras, Rosario Vicente, Raquel Ferrandis, Silvia Polo Martín, Azucena Pajares Moncho, Ignacio Moreno Puigdollers, Juan Pérez Artacho Cortés, Ana Moret Calvo, Ana Pi Peña, María Catalán Fernández, Marina Varela, Pilar Díaz Parada, Raquel Rey Carlín, Sarra Barreiro Aragunde, María Isabel Forés Chiva, A. Javier Agulló, Antonio Pérez Ferrer, María Galiana, Antoni Margarit, Válerie Mourre del Rio, Eva Heras Muxella, Anna Vidal

**Affiliations:** 1 Department of Anaesthesiology and Intensive Care, Clinica Universidad de Navarra, Pio XII, 36, 31008 Pamplona, Spain; 2 Department of Preventive Medicine and Public Health, Medical School, University of Navarra, Pamplona, Spain; 3 Medical Intensive Care Unit, Hospital Clinic, Institut D’investigacio August Pi i Sunyer (IDIBAPS), University of Barcelona, Barcelona, Spain; 4 Department of Anesthesiology and Critical Care, Hospital del Ramon y Cajal, Madrid, Spain; 5 Department of Anesthesiology and Critical Care, Hospital de Cruces, Barakaldo, Vizcaya Spain; 6 Ubikare Technology, Vizcaya, Spain; 7 CIBER de Enfermedades Respiratorias, Instituto de Salud Carlos III, Madrid, Spain; 8 Li Ka Shing Knowledge Institute, St Michael’s Hospital, Toronto, ON Canada; 9 Multidisciplinary Organ Dysfunction Evaluation Research Network, Research Unit, Hospital Universitario Dr. Negrin, Las Palmas de Gran Canaria, Spain; 10 Department of Anesthesiology and Critical Care, Hospital Clinic, Institut D’investigacio August Pi i Sunyer, Barcelona, Spain

**Publisher Correction: Crit Care (2021) 25, 2** 10.1186/s13054-020-03422-3

Following publication of the original article [1], the authors identified errors in the authorship which was caused by the publisher. Collaborating authors part of the COVID-19 Spanish ICU Network were missing in the HTML version of the article while available in the Acknowledgements section.

The authorship has been updated in this Publisher Correction article and the original article [1] has been corrected. The publisher apologises to the authors and readers for the inconvenience caused by this mistake.
