# Supplementary material for: Early corticosteroids are associated with lower mortality in critically ill patients with COVID-19: a cohort study
Source: Crit Care. 2021 Jan 4;25:2. doi: 10.1186/s13054-020-03422-3 (PMC7780210; doi:10.1186/s13054-020-03422-3)
Supplement: Supplementary file 1 — Additional file 1. Methods, Tables S3–6, Figures S2-5 and References. [file 13054_2020_3422_MOESM1_ESM.docx]

**Additional File**

| **Table of contents** | **Page** |
| --- | --- |
| 1. **Methods** | 2 |
| 1. **Tables and Figures** |  |
| **Early use of corticosteroids pre-ICU or first 48h after ICU admission (Figure S2)** |  |
| Figure S2. Kaplan-Meier estimates of mortality according to early use of corticosteroids pre-ICU or within the first 48h after ICU admission. | 5 |
| **Low vs. moderate-high doses of corticosteroids (Tables S3-4, Figure S3)** |  |
| Table S3. Baseline characteristics of patients who received early low or moderate-high doses of corticosteroids | 7 |
| Table S4. Outcomes according to the use of early low or moderate-high doses of corticosteroids | 8 |
| Figure S3. Kaplan-Meier estimates of mortality according to the use of early low or moderate-high doses of corticosteroids | 9 |
| **Ever (early + delayed) vs. never corticosteroids (Tables S5-6, Figure S4)** |  |
| Table S5. Baseline characteristics of patients who Ever or Never received corticosteroids | 10 |
| Table S6. Outcomes depending on whether patients received corticosteroids (Ever or Never) | 11 |
| Figure S4. Kaplan-Meier estimates of 7 days mortality after ICU admission according to the use of early, late or never corticosteroids | 12 |
| 1. **References** | 13 |

1. **Methods**

A total of 882 patients were included in the present study. Out of them, 191 (21.7%) did not receive corticosteroids during total ICU stay, and 691 (78.3%) received corticosteroids during total ICU stay. Considering only the first 48 hours of ICU stay, 485 (55.0%) patients received corticosteroids during this period and 397 (45.0%) patients did not. Out of the 397 patients who were not treated with corticosteroids during the first 48 hours of ICU stay, 206 received corticosteroids afterwards. All patients treated with corticosteroids before ICU admission (n=113) were also treated during ICU stay except for 4 patients.

For some patients, information about type of corticosteroids and dose administered during the first 48 hours is not available (n=23). Out of 485 patients treated within the first 48 hours (462 with dose information) 131(28.4%) patients received low doses and 331 (71.6%) received moderate-high doses.

For the analyses of type of corticosteroids, 11 patients with both dexamethasone and methylprednisolone were excluded. Out of the remaining 871 patients, 23 had no information on the type of drug and 6 were treated with prednisone and were also excluded. Out of 842 patients, 397 were not treated with corticosteroids during the first 48 hours of ICU stay, 106 were treated exclusively with dexamethasone, and 339 were treated exclusively with methylprednisolone.

**Outcome definitions**

Medical and infectious complications followed a standard definition.

***-Medical complications:***

1. Acute respiratory distress syndrome. According to Berlin definition [1]

Mild: PaO_2_/FiO_2_ between 200-300

Moderate: PaO_2_/FiO_2_ between 100-200

Severe: PaO_2_/FiO_2_ < 100

2. Pulmonary hypertension [2]

clinical signs + (imaging or invasive hemodynamic monitor.)

- Ultrasound Right ventricle failure

-Swan Ganz: mean pulmonary artery pressure ≥ 25 mmHg, Pulmonary Artery Wedge Pressure ≤ 15 mmHg, Right Ventricular Pressure > 3 UW.

3. Shock. [3]

Shock is best defined as a life-threatening, generalized form of acute circulatory failure associated with inadequate oxygen utilization by the cells. It is a state in which the circulation is unable to deliver sufficient oxygen to meet the demands of the tissues, resulting in cellular dysfunction. The result is cellular dysoxia, i.e. the loss of the physiological independence between oxygen delivery and oxygen consumption, associated with increased lactate levels. Some clinical symptoms suggest an impaired microcirculation, including mottled skin, acrocyanosis, slow capillary refill time, and an increased central-to-toe temperature gradient.

4. Bleeding [4]

Any sign of hemorrhage plus hemoglobin drop > 3 g/dL or any transfusion with overt bleeding.

5. Pulmonary embolism.[5]

Clinical probability + imaging + laboratory variables.

6. Cardiac arrest.

Pulseless electrical activity or ventricular fibrillation /pulseless ventricular tachycardia

7. Heart failure. [6]

Clinical signs and symptoms with or without preserved left ventricular ejection fraction.

Abnormality of cardiac structure or function leading to failure of the heart to deliver oxygen at a rate commensurate with the requirements of the metabolizing tissues, despite normal filling pressures

8. Arrhythmias.

Alteration from basal electrocardiography or new onset:

- Atrial fibrillation, atrial flutter.

- other supraventricular tachycardia

- Ventricular tachycardia/ Torsade de Pointes

- Atrioventricular block>2 degree.

9. Stroke.[7]

Neurological dysfunction caused by focal cerebral, spinal or retinal infarction ± imaging (caused by ischemia or hemorrhage)

10. Acute kidney injury. According to Kidney Disease Improving Global Outcomes definitions [8]

**Stage 2**: one of the following

- Serum creatinine increase 2.0–2.9 times baseline
- Urinary output <0.5 ml/kg/h during two 6 hours blocks

**Stage 3**: one of the following:

- Serum creatinine increase >3 times baseline
- Serum creatinine increases to >4.0 mg/dl (353 µmol/l)
- Initiation of renal replacement therapy
- Urinary output <0.3ml/kg/h for more than 24 hours
- Anuria for more than 12 hours

11. Transaminitis

Defined by elevation x2 times normal values of bilirubin and/or AST/ALT

*-* ***Infectious complications***

1. Respiratory super-infection. [9]

Defined by the presence of a new lung infiltrate plus clinical evidence that the infiltrate is of an infectious origin, which includes the new onset of fever, purulent sputum, leucocytosis, and decline in oxygenation.

2. Pneumonia. [9]

> 5 points CPIS criteria. Temperature, blood leukocytes, tracheal secretions, oxygenation index, and chest X-ray.

3. Bloodstream infection. [10]

Paired quantitative blood cultures (ratio >=3:1) or paired blood cultures for differential time to positivity (>=120min)

4. Urinary infection [11]

Positive urologic culture and infectious signs/symptoms (Fever, leukocytes, acute phase reactants)

5. Septic shock. [12]

Sepsis with vasopressors required to maintain a mean arterial pressure ≥65 mmHg and serum lactate level > 2 mmol l^-1^ in the absence of hypovolemia. [12]

1. **Tables and Figures**

**Figure S2.** Kaplan-Meier estimates of mortality according to early use of corticosteroids pre-ICU or within the first 48h after ICU admission. We compared 3 sub-cohorts: i) pre-ICU (crude mortality 39.8%); ii) after-ICU: early use, in the first 48 h after ICU admission, excluding pre-ICU (mortality 27.4%), and iii) non-early use of corticosteroids, never + late (mortality 40.3%).

The upper graphs are crude estimates.

The lower graphs are inverse probability weighted (IPW) estimates.

**Crude model**

HR (95% CI) vs non-early corticosteroids:

After-ICU admission/First 48h corticosteroids: 0.64 (0.50 to 0.82)

Pre-ICU corticosteroids: 0.99 (0.71-1.37)

**Adjusted model:**

HR (95% CI) vs non-early corticosteroids:

After-ICU admission/First 48h corticosteroids: 0.54 (0.37 to 0.78)

Pre-ICU corticosteroids: 0.31 (0.09-**1.09**)

p-value of comparison between Pre-ICU vs After-ICU/First 48h: **p=0.40**

**Table S3.** Baseline Characteristics of Patients Receiving early low or moderate-high doses of Corticosteroids**.**

|  | **Non-early steroids** | **Low-early dose** | **Moderate-high-early-dose** | **p-value** |
| --- | --- | --- | --- | --- |
| ***Patients demographics and comorbidities*** | | | | |
| N (%) | 397 (46.22%) | 131 (15.25%) | 331 (38.53%) |  |
| Age, years | 61.6 (12.4)/394 | 61.3 (10.9)/130 | 63.6 (10.6)/330 | 0.05 |
| Female (%) | 138/395 (34.94%) | 41/131 (31.30%) | 107/331 (32.33%) | 0.66 |
| Body mass index (kg/m2) | 29.17 (5.40)/258 | 29.38 (5.26)/66 | 29.20 (5.42)/215 | 0.96 |
| Arterial Hypertension | 180/397 (45.34%) | 65/131 (49.62%) | 165/331 (49.85%) | 0.43 |
| Diabetes Mellitus | 90/397 (22.67%) | 28/131 (21.37%) | 76/331 (22.96%) | 0.95 |
| Chronic heart failure | 6/397 (1.51%) | 2/131 (1.53%) | 5/331 (1.51%) | 1.00 |
| Chronic renal failure | 24/397 (6.05%) | 9/131 (6.87%) | 19/331 (5.74%) | 0.87 |
| Asthma | 8/397 (2.02%) | 6/131 (4.58%) | 10/331 (3.02%) | 0.24 |
| COPD | 16/397 (4.03%) | 5/131 (3.82%) | 17/331 (5.14%) | 0.71 |
| Obese | 141/357 (39.50%) | 52/119 (43.70%) | 99/298 (33.22%) | 0.08 |
| Dyslipidemia | 56/397 (14.11%) | 20/131 (15.27%) | 55/331 (16.62%) | 0.62 |
| Cancer | 19/397 (4.79%) | 2/131 (1.53%) | 6/331 (1.81%) | 0.05 |
| ***Laboratory findings*** |  |  |  |  |
| Hematocrit (%) | 38.99 (5.89)/289 | 39.51 (5.61)/68 | 39.39 (5.70)/228 | 0.54 |
| Platelets, 1000/mm^3^ | 227 (108)/305 | 229 (98)/82 | 246 (111)/266 | 0.06 |
| Leukocytes, 10^3^/μL | 8.62 (5.37)/303 | 9.50 (6.13)/81 | 9.43 (6.25)/261 | 0.10 |
| Lymphocytes, μL | 0.79 (0.47)/297 | 0.96 (1.25)/82 | 0.78 (0.54)/261 | 0.63 |
| CRP, mg/dL | 81.51 (105.40)/274 | 69.94 (85.10)/74 | 64.28 (92.98)/257 | **0.02** |
| Lactate, mmol/L | 0.43 (1.01)/230 | 0.34 (0.56)/58 | 0.42 (0.58)/180 | 0.06 |
| Ferritin, ng/mL | 1561 (1641)/106 | 2344 (2291)/25 | 1630 (1543)/147 | 0.07 |
| D- Dimer, ng/mL | 1825 (2094)/230 | 1857 (2265)/66 | 2285 (2585)/215 | 0.24 |
| CRP/lymphocyte ratio | 156 (295)/273 | 107 (174)/74 | 106 (164)/253 | 0.05 |
| IL-6, pg/mL | 163 (290)/25 | 225 (368)/13 | 321 (612)/44 | 0.77 |
| LDH, U/L | 485 (267)/265 | 509 (230)/69 | 479 (213)/244 | 0.39 |
| Procalcitonin, ng/mL | 1.64 (6.30)/194 | 0.94 (2.16)/55 | 1.22 (4.40)/200 | 0.43 |
| Bilirubin, mg/dL | 0.88 (1.62)/245 | 0.78 (0.61)/70 | 0.77 (0.62)/240 | 0.76 |
| AST, U/L | 59.72 (111.01)/291 | 57.26 (48.76)/80 | 57.14 (64.61)/261 | 0.83 |
| Creatinine, mg/dL | 1.07 (0.71)/297 | 1.15 (1.08)/80 | 0.98 (0.48)/262 | 0.59 |
| Urea, mg/dL | 46.26 (28.69)/197 | 44.19 (25.65)/66 | 47.41 (28.18)/193 | 0.72 |
| NTProBNP, pg/mL | 2068 (6380)/46 | 1151 (1109)/7 | 1797 (4054)/43 | 0.24 |
| ***Vital signs*** |  |  |  |  |
| Temperature, ºC | 37.08 (1.10)/309 | 36.87 (1.17)/75 | 36.66 (1.02)/251 | **<0.001** |
| Mean arterial pressure, mmHg | 86.57 (14.70)/303 | 88.33 (15.62)/75 | 85.80 (15.10)/246 | 0.61 |
| Heart rate, bpm | 86.40 (18.60)/312 | 84.68 (21.09)/74 | 82.75 (18.23)/253 | 0.08 |
| SpO_2_, % | 87.74 (10.52)/299 | 88.23 (7.35)/75 | 88.89 (7.38)/251 | 0.65 |
| Respiratory rate, bpm | 26.66 (7.96)/283 | 24.85 (6.55)/72 | 25.03 (6.53)/233 | **0.03** |
| PaO_2_/FiO_2_ | 149.44 (77.09)/254 | 148.50 (68.47)/68 | 153.2 (81.5)/226 | 0.92 |
| ***Severity scores*** |  |  |  |  |
| APACHE II | 13.14 (5.87)/252 | 14.14 (7.28)/86 | 13.64 (6.51)/254 | 0.80 |
| CURB65 | 1.80 (1.20)/138 | 1.94 (1.01)/32 | 1.96 (1.28)/115 | 0.50 |
| SOFA | 5.70 (2.88)/208 | 6.02 (3.36)/51 | 5.44 (2.89)/209 | 0.38 |

COPD: Chronic Obstructive Pulmonary Disease; CRP: C- Reactive Protein; LDH: Lactate dehydrogenase; AST: Aspartate Aminotransferase; NTProBNP: N-terminal pro-brain natriuretic peptide; APACHE: Acute Physiology and Chronic Health Evaluation; CURB65: Confusion, uremia, elevated respiratory rate, hypotension, and aged 65 years or older; SOFA: Sequential Organ Failure Assessment

**Table S4.** Outcomes according to the use of early low or moderate-high doses of corticosteroids. IPW sample, n=386

|  | **Non early steroids** | **Low-early dose** | **p-value** | **Moderate-high-early dose** | **p-value** |
| --- | --- | --- | --- | --- | --- |
| n (%) | 397 (46.2%) | 131 (15.2%) |  | 331 (38.5%) |  |
| ICU mortality, cases/person-days | 161/24441 | 43/8705 |  | 89/23570 |  |
| ICU mortality (hazard ratio) | 1 (Ref.) | 0.74 (0.51, 1.03) | 0.07 | 0.58 (0.45, 0.75) | **<0.001** |
| ICU mortality (hazard ratio) IPW sample, non-weighted* | 1 (Ref.) | 0.69 (0.35, 1.35) | 0.28 | 0.55 (0.36, 0.85) | **0.008** |
| ICU mortality (hazard ratio) IP-weighted* | 1 (Ref.) | 0.54 (0.21, 1.12) | 0.11 | 0.54 (0.34, 0.80) | **0.007** |
| 7-day mortality, cases/person-days | 37/1873.97 | 9/634 |  | 19/1616 |  |
| 7-day mortality (hazard ratio) | 1 (Ref.) | 0.64 (0.31, 1.31) | 0.22 | 0.53 (0.31, 0.91) | **0.02** |
| 7-day mortality (hazard ratio) IPW sample, non-weighted* | 1 (Ref.) | 0.26 (0.03, 2.010) | 0.19 | 0.52 (0.23, 1.24) | 0.14 |
| 7-day mortality (hazard ratio) IP-weighted* | 1 (Ref.) | 0.17 (0, 0.683) | 0.09 | 0.62 (0.13, 1.44) | 0.30 |
| ICU length of stay, days | 19.67 (15.92) | 18.6 (13.2) |  | 15.7 (12.2) |  |
| ICU length of stay (mean difference) | 0 (Ref.) | -0.59 (-3.37, 2.18) | 0.67 | -3.54 (-5.58, -1.50) | **0.001** |
| ICU length of stay (mean difference) IPW sample, non-weighted* | 0 (Ref.) | -4.21 (-9.24, 0.81) | 0.10 | -4.91 (-8.08, -1.75) | **0.002** |
| ICU length of stay (mean difference) IP-weighted* | 0 (Ref.) | -5.05 (-9.29, -0.93) | 0.01 | -5.38 (-8.64, -2.11) | **0.002** |
| ICU length of stay among survivors, days | 20.18 (16.56) | 19.92 (14.5) |  | 15.3 (12.5) |  |
| ICU length of stay among survivors (mean difference) | 0 (Ref.) | -0.08 (-3.65, 3.47) | 0.96 | -4.62 (-7.21, -2.02) | **0.001** |
| ICU length of stay among survivors (mean difference) IPW sample, non-weighted* | 0 (Ref.) | -4.9 (-10.81, 0.90) | 0.09 | -5.63 (-9.34, -1.93) | **0.003** |
| ICU length of stay among survivors (mean difference) IP-weighted* | 0 (Ref.) | -5.7 (-10.24, -1.16) | 0.02 | -5.73 (-9.79, -1.98) | **0.005** |
| Ventilator-free days | 7.0 (9.1) | 7.6 (8.7) |  | 10.6 (9.5) |  |
| Ventilator-free days (mean difference) | 0 (Ref.) | 0.72 (-1.08, 2.53) | 0.43 | 3.74 (2.41, 5.08) | **<0.001** |
| Ventilator-free days (mean difference) IPW sample, non-weighted* | 0 (Ref.) | 1.44 (-1.68, 4.56) | 0.36 | 3.90 (1.93, 5.86) | **<0.001** |
| Ventilator-free days (mean difference) IP-weighted* | 0 (Ref.) | 2.6 (-0.78, 5.64) | 0.12 | 4.00 (2.05, 5.87) | **<0.001** |
| Medical complications, n (%) | 383 (96.4%) | 127 (96.9%) |  | 327 (98.7%) |  |
| Medical complications (odds ratio) | 1 (Ref.) | 1.09 (0.35, 3.38) | 0.87 | 2.81 (0.91, 8.64) | 0.07 |
| Medical complications (odds ratio) IPW sample, non-weighted* | 1 (Ref.) | 1.48 (0.17, 12.64) | 0.71 | 2.00 (0.49, 8.12) | 0.33 |
| Medical complications (odds ratio) IP-weighted* | 1 (Ref.) | 0.62 (0.10, 1.19) | 0.66 | 1.86 (0.41, 8.57) | 0.39 |
| Infectious complications, n (%) | 236 (59.4%) | 81 (61.8%) |  | 183 (55.2%) |  |
| Infectious complications (odds ratio) | 1 (Ref.) | 1.15 (0.74, 1.73) | 0.47 | 0.88 (0.66, 1.18) | 0.40 |
| Infectious complications (odds ratio) IPW sample, non-weighted* | 1 (Ref.) | 1.16 (0.58, 2.32) | 0.65 | 0.75 (0.49, 1.14) | 0.18 |
| Infectious complications (odds ratio) IP-weighted* | 1 (Ref.) | 1.03 (0.48, 2.34) | 0.92 | 0.78 (0.51, 1.21) | 0.29 |

***N=386**

**Figure S3.** Kaplan-Meier estimates of mortality according to the use of early low or moderate-high doses of corticosteroids. The upper graphs are the crude estimates. The middle graphs are crude estimates in the IP-non-weighted sample (N=386). The lower graphs are IP-weighted estimates (N=386).

**
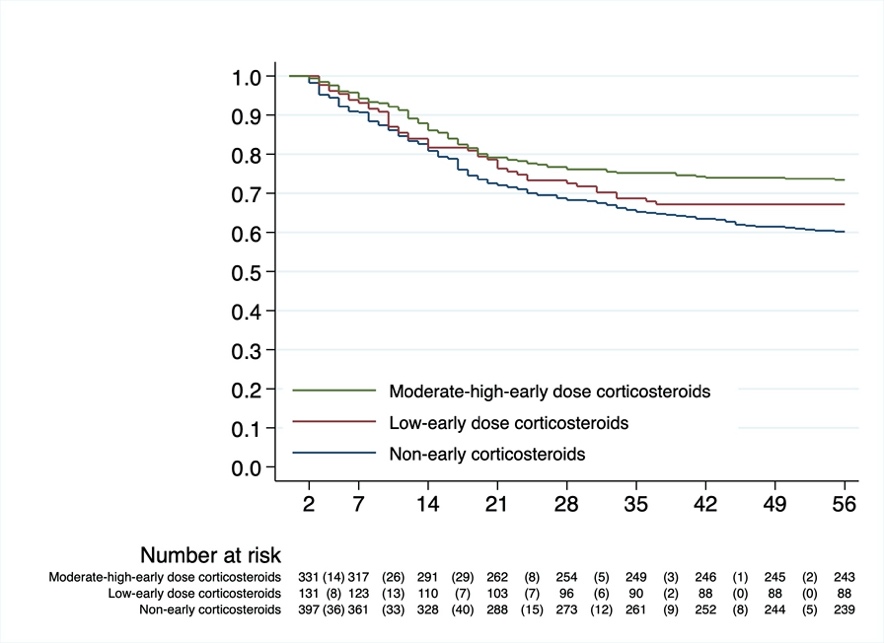
**

**
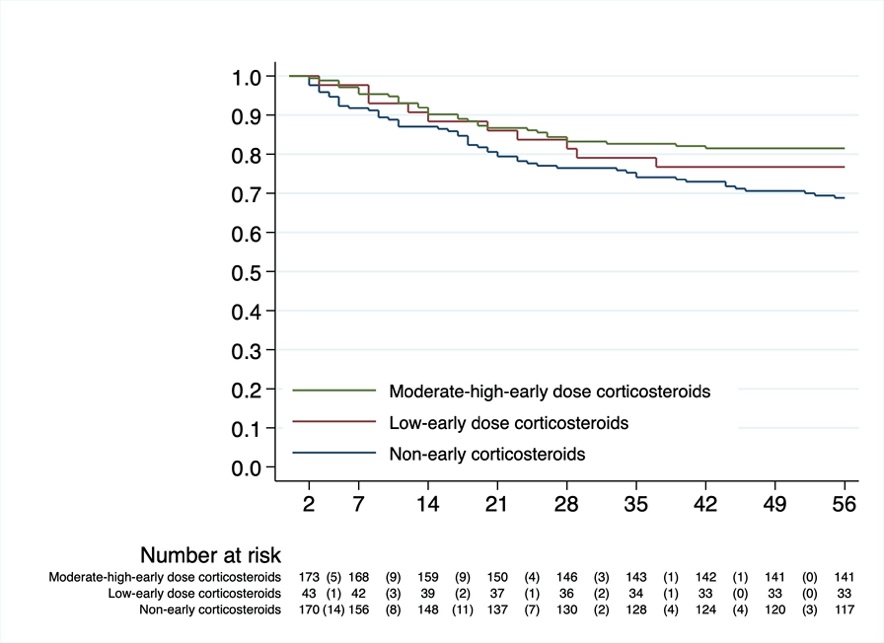
**

**
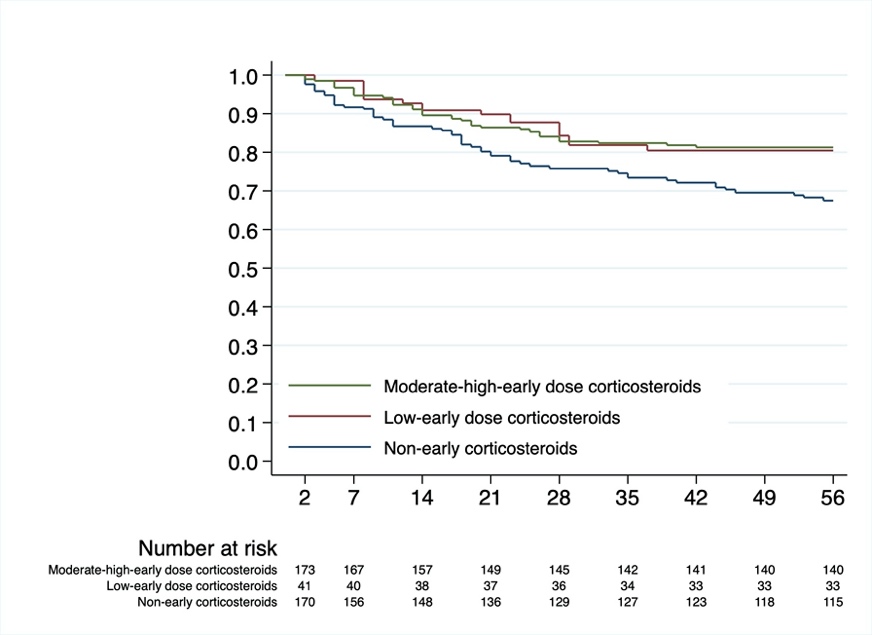
**

**Table S5.** Baseline Characteristics of Patients who have Ever (early + delayed) or Never Received Corticosteroids during hospital stay.

|  | **Never steroids** | **Ever corticosteroids** | **p-value** |
| --- | --- | --- | --- |
| ***Patients demographics and comorbidities*** |  |  |  |
| N (%) | 191 (21.66%) | 691 (78.34%) |  |
| Age, years | 60.0 (13.9)/189 | 62.9 (10.6)/688 | **0.01** |
| Female (%) | 66/191 (34.5%) | 225/689 (32.6%) | 0.66 |
| Body mass index (kg/m2) | 28.71 (5.48)/114 | 29.34 (5.34)/436 | 0.23 |
| Arterial Hypertension | 79/191 (41.36%) | 341/691 (49.35%) | 0.06 |
| Diabetes Mellitus | 43/191 (22.51%) | 158/691 (22.87%) | 1.00 |
| Chronic heart failure | 1/191 (0.52%) | 12/691 (1.74%) | 0.31 |
| Chronic renal failure | 11/191 (5.76%) | 41/691 (5.93%) | 1.00 |
| Asthma | 5/191 (2.62%) | 19/691 (2.75%) | 1.00 |
| COPD | 5/191 (2.62%) | 35/691 (5.07%) | 0.17 |
| Obese | 66/170 (38.82%) | 233/622 (37.46%) | 0.78 |
| Dyslipidemia | 21/191 (10.99%) | 111/691 (16.06%) | 0.08 |
| Cancer | 3/191 (1.57%) | 24/691 (3.47%) | 0.23 |
| ***Laboratory findings*** |  |  |  |
| Hematocrit (%) | 39.13 (6.03)/122 | 39.21 (5.68)/480 | 0.80 |
| Platelets, 1000/mm^3^ | 232.42 (106.60)/130 | 236.25 (108.96)/542 | 0.76 |
| Leukocytes, 10^3^/μL | 8.63 (5.20)/129 | 9.18 (5.95)/534 | 0.20 |
| Lymphocytes, μL | 0.82 (0.45)/126 | 0.82 (0.77)/532 | 0.06 |
| CRP, mg/dL | 85.41 (110.95)/119 | 71.92 (95.77)/504 | 0.25 |
| Lactate, mmol/L | 0.39 (0.53)/99 | 0.44 (1.02)/376 | 0.72 |
| Ferritin, ng/mL | 1744.85 (2191.54)/38 | 1652.27 (1565.18)/247 | 0.61 |
| D- Dimer, ng/mL | 1586.78 (1957.73)/96 | 2125.38 (2407.53)/426 | **0.02** |
| CRP/lymphocyte ratio | 158.09 (342.22)/118 | 124.22 (200.24)/499 | 0.63 |
| IL-6, pg/mL | 269.25 (154.34)/4 | 252.47 (496.98)/83 | 0.08 |
| LDH, U/L | 453.41 (268.91)/114 | 491.48 (231.86)/481 | **0.02** |
| Procalcitonin, ng/mL | 0.91 (2.46)/83 | 1.46 (5.53)/371 | 0.96 |
| Bilirubin, mg/dL | 0.91 (1.46)/98 | 0.80 (1.09)/469 | 0.91 |
| AST, U/L | 68.55 (148.72)/122 | 55.97 (64.22)/528 | 0.72 |
| Creatinine, mg/dL | 1.07 (0.76)/124 | 1.04 (0.66)/534 | 0.67 |
| Urea, mg/dL | 43.73 (35.38)/80 | 47.04 (26.16)/382 | **0.02** |
| NTProBNP, pg/mL | 4505.73 (10334.04)/16 | 1354.94 (3184.04)/80 | 0.22 |
| ***Vital signs*** |  |  |  |
| Temperature, ºC | 37.04 (1.06)/137 | 36.85 (1.09)/517 | **0.02** |
| Mean arterial pressure, mmHg | 86.88 (14.77)/134 | 86.67 (15.43)/510 | 0.87 |
| Heart rate, bpm | 88.18 (19.30)/142 | 84.34 (18.58)/516 | 0.07 |
| SpO_2_, % | 87.66 (10.64)/136 | 88.27 (8.74)/505 | 0.68 |
| Respiratory rate, bpm | 26.69 (8.71)/126 | 25.47 (6.88)/478 | 0.26 |
| PaO_2_/FiO_2_ | 153.67 (78.12)/107 | 149.67 (77.39)/452 | 0.62 |
| ***Severity scores*** |  |  |  |
| APACHE II | 12.45 (5.85)/121 | 13.78 (6.44)/479 | 0.07 |
| CURB65 | 1.67 (1.26)/60 | 1.92 (1.20)/228 | 0.13 |
| SOFA | 5.85 (3.18)/86 | 5.59 (2.88)/388 | 0.43 |

COPD: Chronic Obstructive Pulmonary Disease; CRP: C- Reactive Protein; LDH: Lactate dehydrogenase; AST: Aspartate Aminotransferase; NTProBNP: N-terminal pro-brain natriuretic peptide; APACHE: Acute Physiology and Chronic Health Evaluation; CURB65: Confusion, uremia, elevated respiratory rate, hypotension, and aged 65 years or older; SOFA: Sequential Organ Failure Assessment.

**Table S6. Outcomes according to the use of corticosteroids ever (early + delayed)** **or never during total hospital stay.**

|  | **Whole cohort** | **Never steroids** | **Corticosteroids** | **p-value** |
| --- | --- | --- | --- | --- |
| n (%) | 882 | 191 (21.66%) | 691 (78.34%) |  |
| ICU mortality, cases/person-days | 308/59344 | 70/12274 | 238/47070 |  |
| ICU mortality (hazard ratio) |  | 1 (Ref.) | 0.85 (0.65, 1.11) | 0.25 |
| ICU mortality (hazard ratio) IPW sample, non-weighted* |  | 1 (Ref.) | 0.92 (0.55, 1.53) | 0.75 |
| ICU mortality (hazard ratio) IP-weighted* |  | 1 (Ref.) | 0.69 (0.41, 1.27) | 0.20 |
| 7-day mortality, cases/person-days | 72/5985 | 29/1245 | 43/4740 |  |
| 7-day mortality (hazard ratio) |  | 1 (Ref.) | 0.38 (0.24, 0.62) | **<0.001** |
| 7-day mortality (hazard ratio) IPW sample, non-weighted* |  | 1 (Ref.) | 0.28 (0.12, 0.64) | **0.003** |
| 7-day mortality (hazard ratio) IP-weighted* |  | 1 (Ref.) | 0.23 (0.09, 0.63) | **0.001** |
| ICU length of stay, days | 17.8 (14.2) | 14.2 (13.01) | 18.9 (14.38) |  |
| ICU length of stay (mean difference) |  | 0 (Ref.) | 4.67 (2.41, 6.94) | **<0.001** |
| ICU length of stay (mean difference) IPW sample, non-weighted* |  | 0 (Ref.) | 5.65 (1.77, 9.53) | **0.004** |
| ICU length of stay (mean difference) IP-weighted* |  | 0 (Ref.) | 5.28 (1.32, 9.46) | **0.02** |
| ICU length of stay among survivors, days | 18.0 (14.7) | 15.0 (13.0) | 18.9 (15.1) |  |
| ICU length of stay among survivors (mean difference) |  | 0 (Ref.) | 3.91 (0.96, 6.86) | **0.009** |
| ICU length of stay among survivors (mean difference) IPW sample, non-weighted* |  | 0 (Ref.) | 4.27 (-0.28, 8.83) | 0.06 |
| ICU length of stay among survivors (mean difference) IP-weighted* |  | 0 (Ref.) | 4.48 (0.26, 8.40) | **0.04** |
| Ventilator-free days | 8.4 (9.4) | 9.6 (10.1) | 8.1 (9.1) |  |
| Ventilator-free days (mean difference) |  | 0 (Ref.) | -1.46 (-2.96, 0.04) | 0.06 |
| Ventilator-free days (mean difference) IPW sample, non-weighted* |  | 0 (Ref.) | -2.58 (-5.02, -0.14) | **0.04** |
| Ventilator-free days (mean difference) IP-weighted* |  | 0 (Ref.) | -1.56 (-4.24, 0.96) | 0.28 |
| Medical complications, n (%) | 860 (97.5%) | 179 (93.7%) | 681 (98.55%) |  |
| Medical complications (odds ratio) |  | 1 (Ref.) | 4.56 (1.94, 10.73) | **<0.001** |
| Medical complications (odds ratio) IPW sample, non-weighted* |  | 1 (Ref.) | 3.13 (0.86, 11.41) | 0.08 |
| Medical complications (odds ratio) IP-weighted* |  | 1 (Ref.) | 2.36 (0.51, 10.36) | 0.20 |
| Infectious complications, n (%) | 509 (57.7%) | 92 (48.2%) | 417 (60.35%) |  |
| Infectious complications (odds ratio) |  | 1 (Ref.) | 1.63 (1.18, 2.26) | **0.003** |
| Infectious complications (odds ratio) IPW sample, non-weighted* |  | 1 (Ref.) | 2.31 (1.37, 3.91) | **0.002** |
| Infectious complications (odds ratio) IP-weighted* |  | 1 (Ref.) | 2.38 (1.38, 4.28) | **0.004** |

***N=392.** We used as predictor of treatment covariables measured at ICU admission. For the analysis of early corticosteroids treatment (48 hours) we used as predictor the last available measure of the covariables in the first 48 hours. Therefore, sample size for IPW approach is smaller in this case compare with the early treatment analysis.

**Figure S4.** Kaplan-Meier estimates of 7 days mortality after ICU admission according to the use of early, late or never corticosteroids. The upper graphs are the crude estimates. The middle graphs are crude estimates in the IP-non-weighted sample (N=455). The lower graphs are IP-weighted estimates (N=454).

**
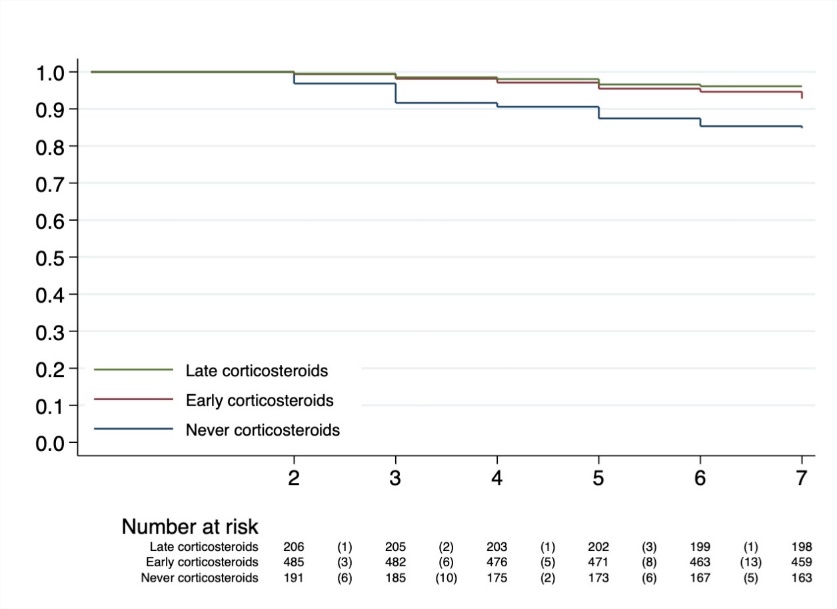

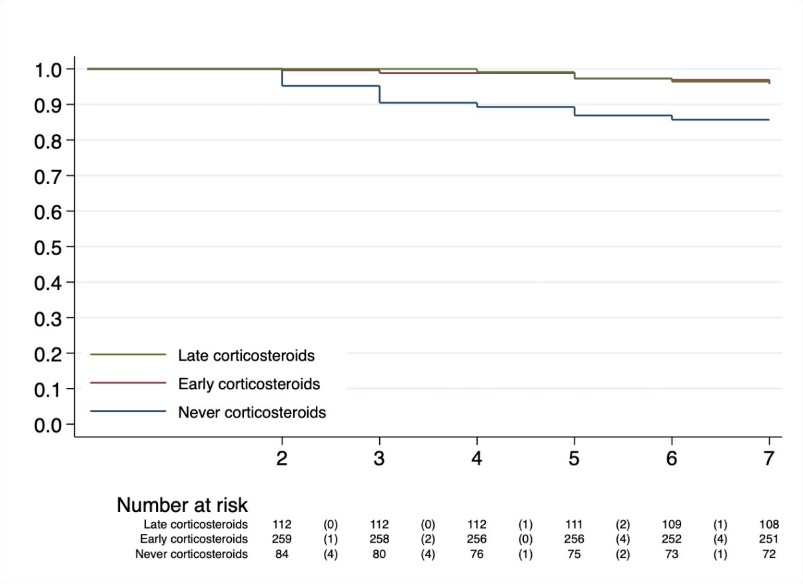

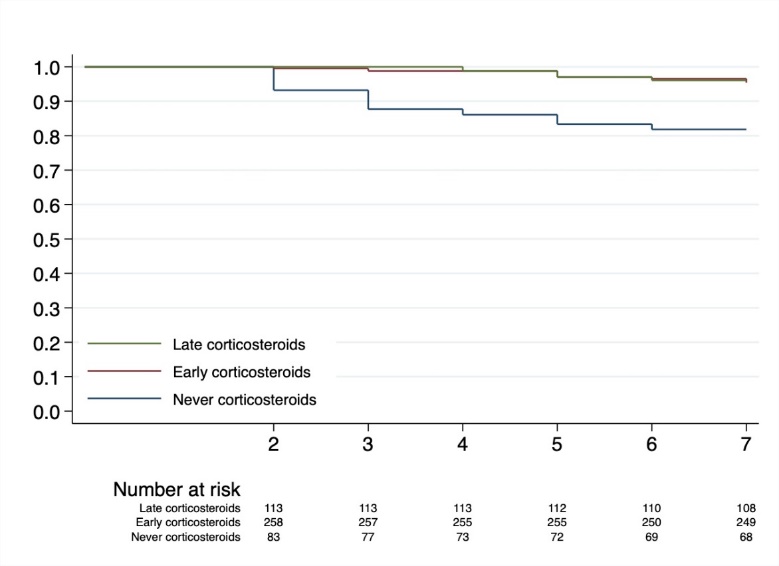
**

**References**

1. Ranieri VM, Rubenfeld GD, Thompson BT, et al. Acute respiratory distress syndrome: The Berlin Definition. JAMA 2012; 307:2526-33.
2. Barberà JA, Román A, Gómez-Sánchez MA, et al. Guidelines on the Diagnosis and Treatment of Pulmonary Hypertension: Summary of Recommendations. Arch Bronconeumol. 2017.
3. Cecconi M, De Backer D, Antonelli M, et al. Consensus on circulatory shock and hemodynamic monitoring. Task force of the European society of intensive care medicine. Intensive Care Med 2014; 40:1795-815.
4. Mehran R, Rao S, Bhatt D, et al. Standardized bleeding definitions for cardiovascular clinical trials. A consensus report from the bleeding academic research consortium. Circulation 2011; 123:2736-47.
5. Konstantinides S, Meyer G, Becattini C, et al. 2019 ESC guidelines for the diagnosis and management of acute pulmonary embolism developed in collaboration with the European respiratory society (ERS). E Respir J 2019, 1901647.
6. McMurray J, Adamopoulos S, Anker S, et al. ESC guidelines for the diagnosis and treatment of acute and chronic heart failure 2012. The task force for the diagnosis and treatment of acute and chronic heart failure 2012 of the European society of cardiology. Developed in collaboration with the heart failure association (HFA) of the ESC. Eur J Heart Fail 2012; 14:803-69.
7. An update definition of the stroke for the 21st century: a statement for healthcare professionals from the American heart association/American stroke. Stroke 2013; 44:2064-89.
8. A European renal best practice (ERBP= position on the kidney disease improving global outcomes (KDIGO) clinical practice guidelines on acute kidney injury: part 1: definitions, conservative management and contrast-induced nephropathy. Nephrol Dial Transplant 2012; 27:4263-72.
9. Singh N, Rogers P, Atwood C, et al. Short-course empiric antibiotic therapy for patients with pulmonary infiltrates in the intensive care unit. Am J Respir Crit Care Med 2000; 162: 505-11.
10. Raad I, Hanna H, Maki D. Intravascular catheter-related infections: advances in diagnosis, prevention and management. Lancet Infect Dis 2007; 7:645-657.
11. Hooton TN, Bradley SF, Cardenas DD, et al. Diagnosis, prevention, and treatment of catheter-associated urinary tract infection in adults: 2009 international clinical practice guidelines from the infectious disease of America. Clin Infect Dis 2010; 50:625-663.
12. Singer M, Deutschman CS, Seymour CW, et al. The third consensus definitions for sepsis and septic shock (sepsis-3). JAMA 2016; 23:801-10.
