# Supplementary material for: Early corticosteroids are associated with lower mortality in critically ill patients with COVID-19: a cohort study
Source: Crit Care. 2021 Jan 4;25:2. doi: 10.1186/s13054-020-03422-3 (PMC7780210; doi:10.1186/s13054-020-03422-3)
Supplement: Supplementary file 2 — Additional file 2. Table S1: Use of corticosteroids and combination of treatments. [file 13054_2020_3422_MOESM2_ESM.docx]

**Table S1. Use of corticosteroids and combination of treatments**

|  | **N (%)** | **Day of start*** | **Duration of treatment*** |
| --- | --- | --- | --- |
| Corticosteroids use (ever during hospital stay) | 691/882 (78.3%) | 0 [0, 2] | 7 [4, 10] |
| Early use of corticosteroids | 485/882 (55.0%) | 0 [0, 0] | 7 [5, 11] |
| -Early use in the first 48 h after ICU admission | 372 (42.2%) |  |  |
| -Onset before ICU admission | 113 (12.8%) |  |  |
| Non-early use of corticosteroids | 397/882 (45.0%) |  |  |
| -Delayed use (after 48 h of ICU admission) | 206 (23.4%) | 6 [3, 10] | 6 [4, 8] |
| -Never used | 191 (21.7%) |  |  |
| ***Timing of corticosteroids administration*** |  |  |  |
| From symptoms onset to first dose (days) |  | 12 [9, 16] / 682*** |  |
| From hospital admission to first dose (days) |  | 4 [2, 8] / 682*** |  |
| From ICU admission to first dose (days) |  | 0 [0, 2] / 691 |  |
| **Systemic corticosteroid drug** |  |  |  |
| *Methylprednisolone* | 516/882 (58.5%) | 0 [0, 4] | 9 [5, 13] |
| *Dexamethasone* | 226/882 (25.6%) | 1 [0, 4] | 9 [5, 12] |
| *Prednisone* | 49/882 (5.6%) | 9 [1, 13] | 3 [2, 7] |
| **Corticosteroid dose** |  |  |  |
| *Low dose* | 142/882 (16.1%) | 0 [0, 4] | 9 [5, 14] |
| *-Early use of low dose* | 131/142 (92.3%) |  |  |
| *-Delayed use of low dose* | 11/142 (7.7%) |  |  |
| *Moderate-high dose* | 526/882 (59.6%) | 0 [0, 4] | 6 [3, 8] |
| *-Early use of moderate-high dose* | 331/526 (62.9%) |  |  |
| *-Delayed use of moderate-high dose* | 195/526 (37.1%) |  |  |
| *Unreported data* | 23/882 (2.6%) |  |  |
|  |  |  |  |
| ***Combinations of treatments*** |  |  |  |
| Only corticosteroids | 344/882 (39.0%) | 0 [0, 3] / 344 | 6 [4, 9] / 344 |
| Tocilizumab | 406/882 (46.0%) | 0 [0, 2] / 406 | 3 [2, 4] / 406 |
| -Only Tocilizumab | 59/882 (6.7%) | 0 [0, 2] / 59 | 3 [2, 4] / 59 |
| -Corticosteroids + Tocilizumab** | 347/882 (39.3%) |  |  |

*Only among treated patients.

**Simultaneously or not.

***For 9 patients there is no information on symptoms onset or day of hospital admission.
