# Supplementary material for: Early corticosteroids are associated with lower mortality in critically ill patients with COVID-19: a cohort study
Source: Crit Care. 2021 Jan 4;25:2. doi: 10.1186/s13054-020-03422-3 (PMC7780210; doi:10.1186/s13054-020-03422-3)
Supplement: Supplementary file 3 — Additional file 3. Table S2: Sensitivity analyses: ICU mortality comparing early versus non-earlyuse of corticosteroids. [file 13054_2020_3422_MOESM3_ESM.docx]

**Table S2.** Sensitivity analyses: ICU mortality comparing early versus non-early use of corticosteroids.

|  | **Total cases/p-day** | **Early vs non-early corticosteroids (HR, IC95%)** | **p-value** |
| --- | --- | --- | --- |
| Main analysis – Crude model | 308/57,589 | 0.71 (0.57, 0.89) | **0.003** |
| Main analysis – IPW sample, non-weighted | 116/33,199 | 0.58 (0.40, 0.84) | **0.003** |
| Main analysis – IP-weighted | 116/33,199 | 0.56 (0.39, 0.79) | **0.002** |
| Main analysis – IP-weighted including missing as a category | 308/57,589 | 0.71 (0.58, 0.88) | **0.003** |
| Additionally, considering mortality during the second day of ICU stay* | 317/60,145 | 0.70 (0.56, 0.88) | **0.002** |
| Corticosteroids use in the first 72 hours (mortality afterwards) | 298/56,717 | 0.78 (0.62, 0.98) | **0.03** |
| Adjusted for tocilizumab use during the first 48h of ICU admission | 308/57,589 | 0.72 (0.58, 0.91) | **0.005** |
| Adjusted for tocilizumab use during total ICU stay | 308/57,589 | 0.71 (0.57, 0.89) | **0.003** |
| Excluding patients with tocilizumab use during total ICU stay | 170/30,806 | 0.61 (0.45, 0.84) | **0.002** |
| Only among patients with invasive mechanical ventilation during the first 48h of ICU stay | 145/26,146 | 0.69 (0.50, 0.96) | **0.03** |
| Only among patients without invasive mechanical ventilation during the first 48h of ICU stay | 163/31,443 | 0.73 (0.54, 0.99) | **0.05** |
| Including corticosteroids use before ICU admission as exposure | 308/57,589 | 0.73 (0.58, 0.91) | **0.006** |
| Excluding patients with use of corticosteroids before ICU admission | 259/50,267 | 0.64 (0.50, 0.82) | **<0.001** |
| **Patients who received corticosteroids within 7 days of symptom onset** | 102/17,383 | 0.80 (0.53, 1.20) | 0.29 |
| Patients who received corticosteroids 7 days or more after symptom onset | 206/40,206 | 0.68 (0.52, 0.90) | **0.007** |
| **Only patients with maximum CRP values in the first 48h ≤10 mg/dL** | 35/11,024 | 0.92 (0.46, 1.83) | 0.81 |
| Only patients with maximum CRP values in the first 48h >10 mg/dL | 185/36,074 | 0.59 (0.44, 0.79) | **<0.001** |
| Patients admitted to ICU before the 9^th^ day from symptoms onset** | 131/23,693 | 0.65 (0.45, 0.94) | **0.02** |
| **Patients admitted to ICU on or after the 9^th^ day from symptoms onset**** | 172/33,199 | 0.75 (0.55, 1.01) | 0.06 |
| Including 149 patients still in ICU excluded for primary analysis | 308/71,298 | 0.71 (0.58, 0.88) | **0.003** |
| Only male patients | 199/38,803 | 0.68 (0.51, 0.90) | **0.006** |
| **Only female patients** | 108/18,676 | 0.79 (0.54, 1.15) | 0.21 |
| **Patients < 60 years old** | 68/23,719 | 0.67 (0.41, 1.08) | 0.09 |
| Patients ≥60 years old | 239/33,489 | 0.69 (0.54, 0.89) | **0.004** |
| Only patients with maximum lymphocytes in the first 48h ≤1 | 179/34,326 | 0.66 (0.49, 0.88) | **0.005** |
| Only patients with maximum lymphocytes in the first 48h >1 | 58/14,825 | 0.59 (0.35, 0.99) | **0.04** |
| Only patients with maximum D Dimer in the first 48h ≤1500 | 82/24,997 | 0.52 (0.34, 0.82) | **0.004** |
| Only patients with maximum D Dimer in the first 48h >1500 | 100/17,112 | 0.71 (0.48, 1.05) | 0.08 |
| Only patients with PaFiO_2_<200 | 131/30,487 | 0.64 (0.45, 0.91) | **0.01** |
| **Only patients with PaFiO_2_≥200** | 31/8826 | 0.61 (0.30, 1.24) | 0.16 |
| Only patients without prevalent hypertension | 144/31,794 | 0.64 (0.46, 0.89) | **0.009** |
| **Only patients with prevalent hypertension** | 164/25,795 | 0.76 (0.56, 1.04) | 0.08 |
| Only patients without type 2 diabetes | 223/45,603 | 0.64 (0.49, 0.83) | **0.001** |
| **Only patients with type 2 diabetes** | 85/11,986 | 0.97 (0.63, 1.48) | 0.88 |
| Only patients without COPD or other pulmonary affections | 291/55,249 | 0.75 (0.59, 0.94) | **0.01** |
| Only patients with COPD or other pulmonary affections | 17/2339 | 0.27 (0.10, 0.72) | **0.009** |
| Only non-oncologic patients | 295/56,098 | 0.72 (0.58, 0.91) | **0.005** |
| **Only oncologic patients** | 13/1491 | 0.66 (0.18, 2.39) | 0.52 |

*Patients who died or were discharged the same day of ICU admission were not considered in any analysis. In this sensitivity analysis, we included patients who either die or were discharged the day after ICU admission.

**The median time from symptoms onset to ICU admission was 9 days.
