# Supplementary material for: Early corticosteroids are associated with lower mortality in critically ill patients with COVID-19: a cohort study
Source: Crit Care. 2021 Jan 4;25:2. doi: 10.1186/s13054-020-03422-3 (PMC7780210; doi:10.1186/s13054-020-03422-3)
Supplement: Supplementary file 4 — Additional file 4. Figure S1: Restricted cubic spline to plot the odds ratio (95% confidenceinterval) of mortality according to the onset day of corticosteroids treatment. [file 13054_2020_3422_MOESM4_ESM.docx]

**Figure S1**. Restricted cubic spline to plot the Odds ratio (95% confidence interval) of mortality according to the onset day of corticosteroids treatment.

Non-treated patients were excluded. Patients with treatment previous to ICU admission are codified as starting day 0.

OR (95% CI) for starting 1 day later corticosteroids treatment:

Crude model: 1.05 (1.02 to 1.08); **p=0.001**

Adjusted model*: 1.09 (1.04 to 1.14); **p<0.001**

* Adjusted for age, sex, APACHE, SOFA, Pa/FiO2, prevalence of type 2 diabetes, and prevalence of hypertension.
